# A Facile Synthetic Route to Amphiphilic Poly(*Meta*-Phenylene Ethynylene) and Poly(*Meta*-Phenylene Ethynylene)-*Block*-Polyisocyanide Using a Single Catalyst

**DOI:** 10.3390/polym10090936

**Published:** 2018-08-23

**Authors:** Chonglong Li, Xunhui Xu, Lei Xu, Na Liu

**Affiliations:** 1 Department of Polymer Science and Engineering, School of Chemistry and Chemical Engineering, and Anhui Key Laboratory of Advanced Functional Materials and Devices, Hefei University of Technology, Hefei 230009, China; lililong0@126.com (C.L.); xuxunhui@mail.hfut.edu.cn (X.X.); 18856036326@163.com (L.X.); 2 School of Chemistry and Chemical Engineering, North Minzu University, Yinchuan 750021, China

**Keywords:** helical polymers, helix inversion, poly(*meta*-phenylene ethynylene), poly(*meta*-phenylene ethynylene)-*block*-polyisocyanide

## Abstract

An optically active, amphiphilic *meta*-phenylene ethynylene (*m*-PE) bearing a chiral amide pendant was designed and synthesized. Living polymerization of *m*-PE using alkyne-Pd(II) as the initiator afforded well-defined poly(*meta*-phenylene ethynylene) (*m*-PPE). These *m*-PPEs were found to have a stable helical conformation in THF, 1,4-dioxane, and CH_3_CN and showed split Cotton effects over the range of 245–400 nm. The positive first Cotton effect was observed at a wavelength of approximately 308 nm, and the negative second Cotton effect was observed at a wavelength of approximately 289 nm. The *m*-PPEs exhibited helical conformational changes in different mixed solvents and showed effective solvent-dependent helix inversion in CHCl_3_/THF solutions. The sign of the Cotton effect of *m*-PPE was inverted at 25 °C by varying the mixing ratio of THF and CHCl_3_. Finally, amphiphilic poly(*meta*-phenylene ethynylene)-*block*-polyisocyanide containing hydrophilic PPE and hydrophobic PPI segments were facilely prepared using Pd(II)-terminated *m*-PPE as the macroinitiator. This block copolymer can self-assemble into well-defined spherical nanostructures in a selective THF/CH_3_OH solution. This efficient polymerization will open up enormous opportunities for the preparation of functional amphiphilic block copolymers in a wide variety of fields.

## 1. Introduction

Helical polymers, such as chiral packing materials for separating enantiomers in chromatography [1,2], asymmetric catalysis [3,4,5,6], and chiral selectors [7,8] have attracted considerable research attention during the past few decades in the fields of polymer chemistry and materials science. To date, many optically active helical polymers have been synthesized by direct polymerization of monomers with the required functional groups. Moore and his collaborators found that *m*-PPEs was able to fold into a compact helical conformation in polar solvents, such as acetonitrile [9,10,11], and were capable of binding small molecules, such as (−)-α-pinene [12]. Therefore, *m*-PPEs have potential for use as chiral and biological materials [13,14].

Currently, *m*-PPEs is one of the most popular structural motifs for foldamers and can assume either a left-handed or right-handed helical conformation, with the helix controlled by either thermodynamics or kinetics. Many *m*-PPEs exist as synergistic dynamic helical foldamers during excess single-handed helix formation; these are chain molecules that have a specific conformational state in solution, and their structures are stabilized through a series of intra- and/or intermolecular noncovalent bonding interactions [15].

The helical reversal states that separate left- and right-handed helical segments are unfavorable in terms of energy. Therefore, helical polymers require energy to convert between left- and right-handed helical conformations. The dynamic helical polymers have a very low helix inversion barrier. Using externally supplied energy, the polymer can overcome the small energy barrier of helical inversion, and it is easy to undergo a helix–helix transition from one helical conformation to another. Therefore, helix inversion can be induced by the application of external stimuli [16,17], such as changing the temperature [18,19,20,21], light [22,23], metal ions [24,25,26], pH [27,28], and solvent composition [29,30,31]. Some studies have reported the results of helix–helix inversion of PPE without the help of extraneous molecules [32].

In this study, amphiphilic *m*-PE bearing a chiral amide pendant was designed and synthesized. Polymerization of *m*-PE afforded a well-defined *m*-PPE, which was achieved via the catalyst-transfer polycondensation mechanism based on Pd(II)-catalyzed Sonogashira coupling reactions [33]. The helical structure of *m*-PPE was rigid and stable, and it had a dynamic folded conformation. Meanwhile, it was found that *m*-PPE formed a preferred-handed helix in the presence of solvents such as CH_3_CN and THF and that helix–helix inversion occurred in THF/CHCl_3_ solutions.

PPEs and their block copolymers have been widely used in explosives detection [34] and molecular wire applications [35,36]. Since polyisocyanide can easily form into a rigid helical conformation, it has been connected to PPE to provide better block copolymers. It is known that the synthesis of poly(*meta*-phenylene ethynylene)-*block*-polyisocyanide has been achieved in previous works using Pd(II)-terminated *m*-PPE as the macroinitiator. These block copolymers can self-assemble into well-defined spherical nanostructures in selective solvents. In the future, amphiphilic block copolymers will provide new materials with interesting self-assembly and optical properties. Thus, the development of novel well-defined block copolymers with stable helical conformation is of great interest.

## 2. Experimental

### 2.1. Chemicals and Reagents

All solvents were obtained from Sinopharm. Co. Ltd. (Beijing, China). and were distilled under reduced pressure prior to use. All chemicals were purchased from Aladdin (Shanghai, China), Sinopharm (Beijing, China), and Sigma-Aldrich Chemical Co. Ltd. (St. Louis, MO, USA) and were used as received without further purification unless noted otherwise. Phenylene ethynylene monomer **1** was prepared according to the procedures reported by Ramakrishnan [37] and Yashima [38]. The Pd(II) initiator and poly-**1**_m_ were prepared according to procedures previously reported by our group [39,40,41]. All structures were confirmed by ^1^H NMR spectra, ^13^C NMR, and FT-IR. 

### 2.2. Instrumentation and Characterization

^1^H and ^13^C NMR spectra were recorded using a 600 MHz Bruker (Bruker, Fremont, CA, USA). FT-NMR spectrometer (600 MHz, or 150 MHz, respectively). Size exclusion chromatography (SEC) was performed with a Waters 1515 pump and Waters 2414 differential refractive index (RI) detector (Waters, Milford, MA, USA) (set at 40 °C) using a series of two linear TSK gel GMHHR-H columns. Molecular weight (*M*_n_) and polydispersity (*M*_w_/*M*_n_) data were reported relative to those of the polystyrene standards. Tetrahydrofuran (THF) was used as the eluent at a flow rate of 0.8 mL/min. FT-IR spectra were recorded with a Perkin–Elmer Spectrum (Thermo Nicolet, Waltham, MA, USA) BX FT-IR system using KBr pellets at 25 °C. Circular dichroism (CD) and UV-vis absorption spectra were obtained using a JASCO J1500 spectropolarimeter (JASCO, Tokyo, Japan) and UNIC 4802 UV/VIS double beam spectrophotometer (Unico, Dayton, NJ, USA), respectively. Atomic force microscopy (AFM) images were acquired at room temperature in the tapping mode with a Digital Instruments Dimension 3100 Scanning Probe Microscope (Bruker, Karlsruhe, Germany). Transmission electron microscopy (TEM) was performed on a JEM-2100F operating at an accelerating voltage of 200 kV (JEOL, Tokyo, Japan).

### 2.3. Representative Polymerization Procedure for PPE (Poly-***1***_25_)

A 10 mL oven-dried flask was charged with triphenylphosphine (PPh_3_) (3.41 mg, 13.0 µmol) and a stirring bar. A solution of bis(triethylphosphine)-((4-methoxyphenyl)ethynyl) palladium(II) chloride (1.33 mg, 2.62 µmol) in dry THF (0.3 mL) was then added under dry nitrogen. After the reaction flask was stirred at 30 °C for 3 h, copper iodide (CuI) (2.48 mg, 13.0 µmol), a solution of monomer **1** (31.16 mg, 65.6 µmol) in dry THF (0.3 mL), and Et_3_N (0.2 mL) were added under a dry nitrogen atmosphere. The reaction flask was then immersed in an oil bath and stirred at 55 °C for 10 h. The progress of the polymerization was monitored by SEC until the molecular weight of the yielded poly-**1**_25_ ceased to increase. After cooling to room temperature, the polymerization solution was precipitated in a large amount of *n*-hexane, collected by centrifugation, and dried in vacuum at room temperature overnight to afford the desired poly-**1**_25_ (15.94 mg, 70%). SEC: *M*_n_ = 6.38 kDa, *M*_w_/*M*_n_ = 1.17. ^1^H NMR (600 MHz, CDCl_3_, 25 °C) δ: 8.05–7.90 (br, 2H), 7.82–7.72 (br, 1H), 7.54–7.45 (br, 1H), 3.80–3.50 (br, 12H), 3.46–3.41 (br, 2H), 3.32–3.29 (br, 1H), 3.29–3.24 (br, 3H), 1.24–1.19 (br, 3H).

### 2.4. Representative Copolymerization Procedure for PPE-b-PPI (poly(***1***_25_-b-***2***_60_))

Monomer **2** (24.16 mg, 84 µmol) was added to a solution of freshly generated Pd(II)-terminated PPE macroinitiator (poly-**1**_25_, *M*_n_ = 6.38 kDa, *M*_w_/*M*_n_ = 1.17), and the reaction mixture was stirred at 55 °C for 12 h ([**2**]0 = 0.1 M, [**2**]0/[Pd]0 = 60/1). When the molecular weight ceased to increase according to SEC, the solution was quenched by precipitation into a large amount of CH_3_OH. The precipitated solid was collected by filtration and washed with cold THF and CH_3_OH to afford the desired block copolymer poly(**1**_25_-*b*-**2**_60_) (34.54 mg, 79% yield over the two steps) as a yellow solid. SEC: *M*_n_ = 21.5 kDa, *M*_w_/*M*_n_ = 1.20. ^1^H NMR (600 MHz, CDCl_3_, 25 °C): δ 7.09–8.02 (br, 6H, ArH of PPE and PPI segment), 5.51–6.05 (br, 2H, ArH of PPI segment), 4.42–3.27 (br, 20H, OCH_2_ of PPE and CO_2_CH_2_ of PPI segment of PPE segment), 1.97–0.87 (br, 22H, CH_3_, alkyl chains of PPI segments).

## 3. Results

### 3.1. Homopolymerization of m-PPE and Its Chiroptical Properties

#### 3.1.1. Synthesis and Polymerization of Optically Active *m*-Phenylene Ethynylenes

The optically active monomer **1** was synthesized according to literatures reported by Ramakrishnan and Yashima, as outlined in Scheme S1.

**Scheme 1:**
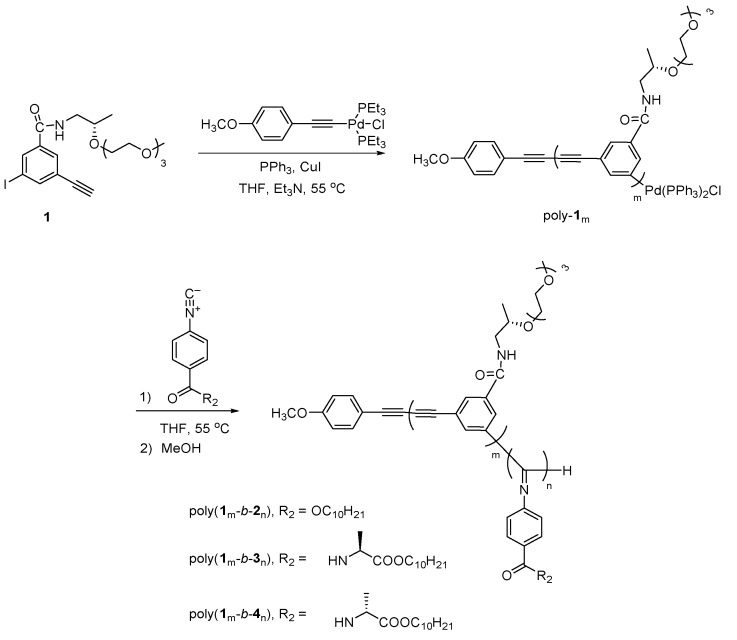
Synthesis of the Poly-**1**_m_ Homopolymer and PPE-*b*-PPI block copolymer.

We used the Pd(II) initiator to prepare amphiphilic conjugated polymer *m*-PPE. As shown in Scheme 1, polymerization of monomer **1** was carried out in THF at 55 °C using the Pd(II) complex and triethylamine in the presence of PPh_3_ and CuI under a dry nitrogen atmosphere ([**1**]0/[Pd]0 = 25, [PPh3]0/[**1**]0 = 0.2, [**1**]0 = 0.12 M). The reaction mixtures were stirred in THF at 55 °C for 10 h. When the molecular weight of the polymer ceased to increase, the reaction was quenched with *n*-hexane and separated by centrifugation. We successfully synthesized the desired homopolymer poly-**1**_m_ (the subscript indicates the initial feed ratio of the monomer to initiator, the same notation is used below) as confirmed by SEC analysis. The *M*_n_ and *M*_w_/*M*_n_ of synthetic poly-**1**_25_ were determined up to 6.38 kDa and 1.17, respectively, by SEC analysis with polystyrene standards (Table 1). ^1^H NMR spectra of the isolated poly-**1**_25_ were measured in CDCl_3_ at room temperature (Figure S19, Supplementary Materials).

**Figure 1:**
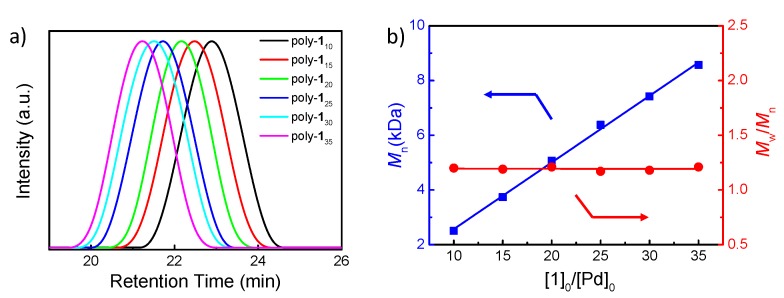
(**a**) Size exclusion chromatography (SEC) chromatograms of poly-**1**_m_ prepared via polymerization of monomer **1** using the Pd(II) catalyst in THF with different initial feed ratios of monomer to catalyst. (**b**) Plots of the *M*_n_ and *M*_w_/*M*_n_ values of poly-**1**_m_ with the initial feed ratio of monomer **1** to the Pd(II) initiator. *M*_n_ and *M*_w_/*M*_n_ were determined by SEC with the polystyrene standard (SEC conditions: eluent = THF, temperature = 40 °C).

A series of polymerizations of monomer **1** using the Pd(II) initiator were carried out with different initial feeding ratios of monomer **1** to the initiator in THF at 55 °C (Figure 1a). As expected, all of the isolated polymers were isolated at high yields (>65%), and the molecular weight of the polymer gradually increased with the increase of the initial feed ratio of [**1**]_0_/[Pd]_0_. Moreover, as shown in Figure 1b, the *M*_n_s values of the isolated poly-**1**_m_ were linearly correlated to the initial feed ratio of [**1**]_0_/[Pd]_0_. All of the polymer dispersity values were narrowly distributed with *M*_w_/*M*_n_ < 1.30 (Table 1). The structure of the isolated poly-**1**_m_ was further verified by ^1^H NMR analysis.

#### 3.1.2. Chiroptical Properties in Dilute Solution

Ester-bound *m*-PPEs folded into helical conformations in acetonitrile and adopted random-coil conformation in CHCl_3_ [12]. In the UV-vis spectra of Ester-bound *m*-PPEs, the ratio of the UV maximum absorbances at 303 and 289 nm showed that the foldamers had almost the same relative intensity and were in a randomly oriented conformation in a mixture of the cisoid and transoid states in CHCl_3_ [9,12]. Oligomers with low values of *A*_303_/*A*_289_ had a high degree of folding [9]. In acetonitrile, on the other hand, a decrease of *A*_303_/*A*_289_ in the UV spectra was observed [42,43]. Yashima and coworkers previously reported that poly(meta-phenylene ethynylene)s bearing l-alanine-derived oligo(ethylene glycol) side chains ((*S*)-PPEa) had a preferred-handed helical conformation induced by the covalent bonding of chiral alanine pendants, even in CHCl_3_ [38]. In CHCl_3_, the helical conformation of (*S*)-PPEa is likely to be stabilized by intramolecular hydrogen bonds between nonadjacent amide pendants [38]. 

**Figure 2:**
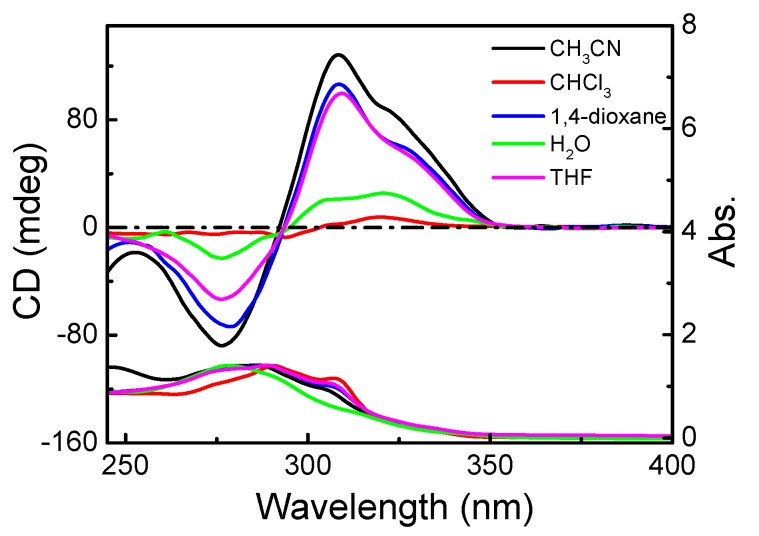
CD and UV spectra of poly-**1**_25_ measured in different solvents at 25 °C (c = 0.04 mg/mL).

To compare the folding properties of poly-**1**_25_, the chiroptical properties were investigated in different solvents at 25 °C by CD (Figure 2). It was observed that poly-**1**_25_ underwent a solvent-dependent conformational transition from an approximately random-coil conformation in CHCl_3_ to a helical structure in acetonitrile. Poly-**1**_25_ showed similar split Cotton effects in THF, 1,4-dioxane, and H_2_O over the range of 245–400 nm, i.e., the positive first Cotton effect occurred at a wavelength of approximately 308 nm and the negative second Cotton effect occurred at a wavelength of approximately 289 nm. However, poly-**1**_25_ showed a negligible CD signal in CHCl_3_, indicating that the intramolecular hydrogen bonds in the poly-**1**_25_ chains might interfere with the chiral induction of the main chain with the chiral amide pendants and formed the helical structure of poly-**1**_25_ with lower regularity. The CD spectra of poly-**1**_25_ showed a positive first Cotton effect at 308 nm at 25 °C in CH_3_CN, which was a poor solvent for the main chain, but a good solvent for the sidechain. In THF and 1,4-dioxane, the interior stress in the polymer was relaxed and the chiral amide pendant was able to be efficiently induced to afford stronger CD absorptions of poly-**1**_25_.

**Figure 3:**
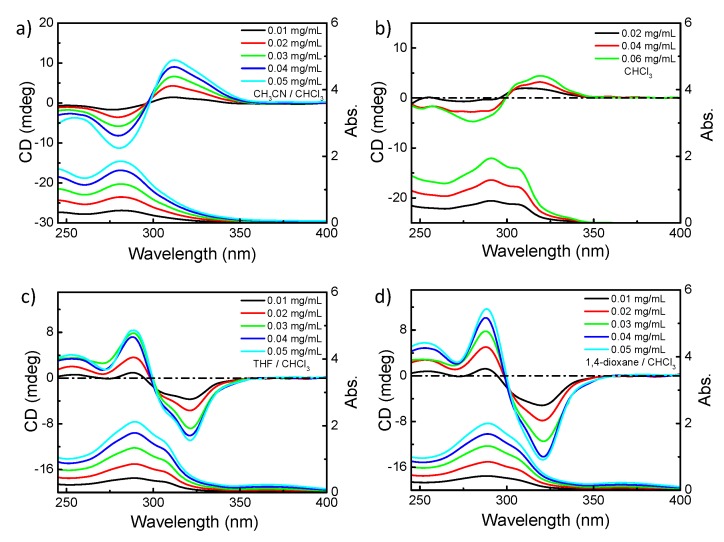
CD and UV-vis spectra of poly-**1**_25_ in mixed solvents containing 5% of CHCl_3_ by volume at 25 °C in various concentrations. (**a**) CH_3_CN and CHCl_3_. (**b**) CHCl_3_. (**c**) THF and CHCl_3_. (**d**) 1,4-dioxane and CHCl_3_.

The CD and UV-vis spectra of poly-**1**_25_ were measured to characterize the chiroptical properties of optically active *m*-PPE. We have found an interesting phenomenon that when 0.15 mL of a CHCl_3_ solution of poly-**1**_25_ (1.0 mg/mL) was added to 2.85 mL of THF, a completely different response was observed in THF/CHCl_3_ solutions with a 5% volume of CHCl_3_. As shown in Figure 3c, poly-**1**_25_ had a negative CD signal at approximately 308 nm and a positive CD signal at a wavelength of approximately 289 nm in the THF/CHCl_3_ solvents. The Cotton effect of poly-**1**_25_ in THF/CHCl_3_ was opposite in sign to that in pure THF at the same concentration, indicating that the polymer assumed helical conformations with reversed senses in the mixture solvents. Upon the addition of 0.15 mL of a CHCl_3_ solution containing poly-**1**_25_ at various concentrations to 1,4-dioxane, we found that poly-**1**_25_ assumed a helical conformation with reversed senses in 1,4-dioxane/CHCl_3_ solutions containing 5% of CHCl_3_ by volume compared to pure 1,4-dioxane (Figure 3d). However, upon addition of 0.15 mL of a CHCl_3_ solution containing poly-**1**_25_ at various concentrations to CH_3_CN, we found that poly-**1**_25_ assumed the same helical conformations as that those in the pure CH_3_CN solution (Figure 3a).

**Figure 4:**
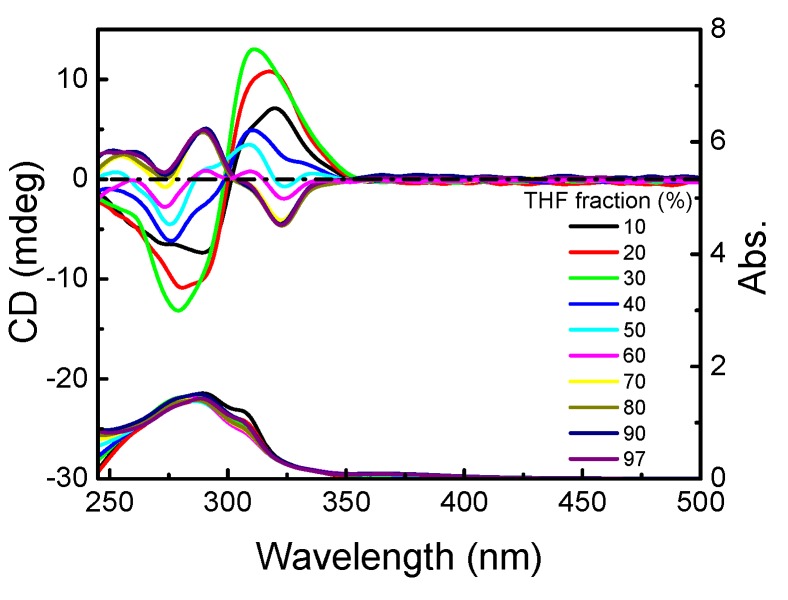
CD and UV-vis spectra of poly-**1**_25_ measured in THF/CHCl_3_ solutions with different THF fractions at the concentration of 0.03 g/L.

To compare the helix inversion properties of poly-**1**_25_, the chiroptical properties were studied at different ratios of THF to CHCl_3_ at 25 °C by CD spectroscopy UV-vis spectra (Figure 4). Therefore, it was expected that the helical inversion occurred according to the change of the solvent composition between CHCl_3_ and THF. Adding 0.09 mL of a CHCl_3_ solution (1.0 mg/mL) containing poly-**1**_25_ to different ratios of THF and CHCl_3_ at a concentration of 0.03 g/L, we further investigated the effect of the solvents on the poly-**1**_25_ CD and UV-vis spectral patterns. The polymer exhibited a positive first CD signal and an intense UV-vis absorption peak at 308 nm in THF/CHCl_3_ solutions with a 10% THF fraction (Figure 4). As the percentage of THF in CHCl_3_ increased from 10% to 30%, the positive first Cotton effect of poly-**1**_25_ increased steadily and the CD signals were still positive at 308 nm. When THF reached 40%, the first CD signal intensity suddenly decreased. Upon further increasing the THF amount to 50%, the portion of the CD signals at 322 nm inverted to the opposite signs and had a negative value. The CD spectra intensity at 322 nm in a 70% THF solution showed the largest negative value, which was almost identical to that in a 97% THF solution. The positive first CD signal at 308 nm gradually transformed into a negative CD signal with the increase of the THF volume in the THF/CHCl_3_ mixtures.

It was observed from these results that the poly-**1**_25_ changed from a helix structure with a predominantly single-handed helix to a helix structure with the opposite screw sense; this change did not occur via the random coil structure, but rather through a direct helix–helix transformation with the change of the THF/ CHCl_3_ composition. The helical transition from one predominant handedness to another spanning a wide composition range might be caused by the competitive interactions between the molecules of the two solvents of THF and CHCl_3_ with the amide chains.

It is considered that the conformations of the polymers at different ratios of THF to CHCl_3_ are energetically favorable to assume one-handed helices with positive or negative CD signals. Such transitions are thought to result from the entropy difference between the left- and right-handed helices produced by changes in the structure of the main chains and side chains, or changes in the solvation, or changes intra- and intermolecular hydrogen bond interactions; however, the exact mechanism of this transition is still unclear.

### 3.2. One-Pot Synthesis of a PPE-b-PPI Block Copolymer and Chiroptical Properties

**Figure 5:**
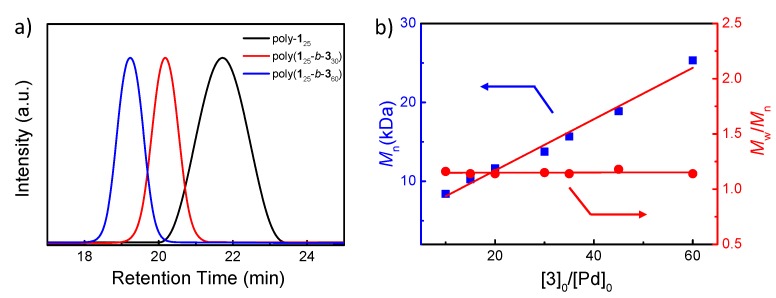
(**a**) SEC of poly-**1**_25_, poly(**1**_25_-*b*-**3**_30_) and chain extended poly(**1**_25_-*b*-**3**_60_) performed by copolymerization of **3** with Pd(II)-terminated poly(**1**_25_-*b*-**3**_30_) as the macroinitiator ([**3**]_0_/[Pd]_0_ = 30). (**b**) Plots of the *M*_n_ and *M*_w_/*M*_n_ values of poly(**1**_m_-*b*-**3**_n_)s with the initial feed ratios of monomer **3** to Pd(II)-terminated poly-**1**_25_ (*M*_n_ = 6.38 kDa, *M*_w_/*M*_n_ = 1.17). SEC conditions: temperature = 40 °C; eluent = THF.

According to Scheme 1, polymerization of monomer **1** was carried out using the Pd(II) complex according to the procedure described above ([**1**]0/[Pd]0 = 25, [PPh3]0/[**1**]0 = 0.2, [**1**]0 = 0.12 M). When polymerization of monomer 1 was completed as indicated by SEC, a THF solution of cyanide monomer 3 ([**3**]0 = 0.15 M, [**3**]0/[Pd]0 = 30) was added to the reaction system, and the mixture was stirred at 55 °C for 10 h. When the molecular weight of the polymer ceased to increase, the reaction was quenched by CH3OH. The residue was concentrated, dissolved with a small amount of THF, and poured into CH3OH to precipitate the solid. The yield of poly(**1**_25_-*b*-**3**_30_) was 70% in two steps. The SEC of the obtained poly(**1**_25_-*b*-**3**_30_) was located in the shorter retention-time region compared to that of poly-**1**_25_. The *M*_n_ of poly(**1**_25_-*b*-**3**_30_) was estimated to be 13.8 kDa (*M*_w_/*M*_n_ = 1.10), which was larger than that of poly-**1**_25_ (*M*_n_ = 6.38 kDa, *M*_w_/*M*_n_ = 1.17). To verify the activity of Pd(II)-terminated poly(**1**_25_-*b*-**3**_30_), freshly Pd(II)-terminated poly(**1**_25_-*b*-**3**_30_) was prepared and monomer **3** in THF was added into the system. The *M*_n_ of the resulting poly(**1**_25_-*b*-**3**_60_) was determined to be 25.3 kDa, larger than that of poly(**1**_25_-*b*-**3**_30_) (*M*_n_ = 13.8 kDa, *M*_w_/*M*_n_ = 1.10) (Figure 5a). Taking advantage of the living nature of the block copolymerization, a series of block copolymers was obtained using Pd(II)-terminated poly-**1**_25_ (*M*_n_ = 6.38 kDa, *M*_w_/*M*_n_ = 1.17) as the macroinitiator. All of the block copolymers were isolated at high yields, and the results of the one-pot block copolymerization are summarized in Table 2. The plots of the *M*_n_ and *M*_w_/*M*_n_ values of poly(**1**_m_-*b*-**3**_n_)s with the initial feed ratios of monomer **3** to the macroinitiator, Pd(II)-terminated poly-**1**_25_, are summarized in Figure 5b.

**Figure 6:**
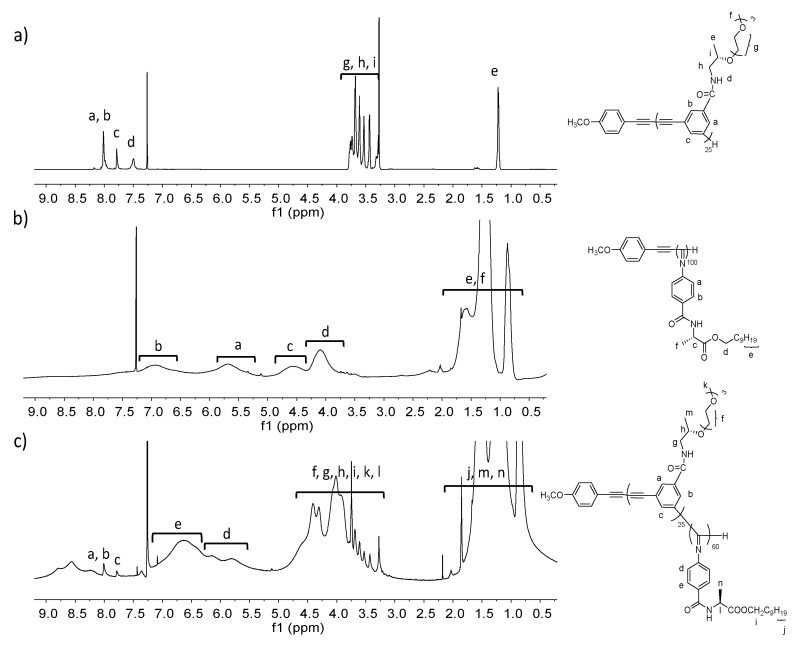
^1^H NMR spectra (600 MHz) of PPE homopolymer poly-**1**_25_. (**a**) PPI homopolymer poly-**3**_100_. (**b**) PPE-*b*-PPI block copolymer poly(**1**_25_-*b*-**3**_60_) (**c**) measured in CDCl_3_ at 25 °C.

To collect detailed structural information, the one-pot synthetic block copolymer poly(**1**_m_-*b*-**3**_n_)s was characterized by FT-IR and NMR measurements. The FT-IR spectra of the polymer are given in Figure S28. More detailed information about the isolated block copolymer poly(**1**_25_-*b*-**3**_60_) was obtained from ^1^H NMR spectroscopy, as shown in Figure 6c.

As shown in Figure 6, proton signals that can be attributed to both PPE and PPI blocks were observed, and were the proton signals of PPE and PPI homopolymers. Monomer **1** exhibited resonance signals at 8.17, 7.89, and 7.29 ppm corresponding to the asymmetric aryl ring, and the signal at 3.30 ppm is attributed to the characteristic signal of the OCH_3_ of the PPE segment (Figure S16, Supplementary Materials). The ^1^H NMR spectra of the obtained poly-**1**_25_ were similar after **1** was polymerized by the Pd(II) initiator (Figure 6a). The spectra showed a singlet resonance at 8.01, 7.78, and 7.50 ppm ascribed to the phenyl ring and a singlet resonance at 3.27 ppm of OCH_3_. The poly(**1**_25_-*b*-**3**_60_) copolymer showed resonances at 8.01, 7.78, 7.50, and 3.27 ppm, corresponding to the aryl and OCH_3_ protons of the PPE segment, and also exhibited resonances at 6.68 and 5.81 ppm, which were assigned to the aryl protons of the PPI segment. Phenyl isocyanide monomers were consumed by SEC and participated in the copolymerization. 

**Figure 7:**
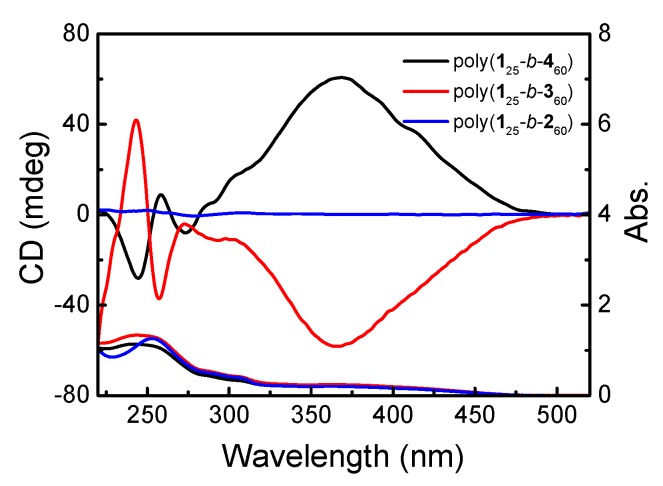
CD and UV-vis spectra of poly(**1**_25_-*b*-**2**_60_), poly(**1**_25_-*b*-**3**_60_), and poly(**1**_25_-*b*-**4**_60_) measured in THF at 25 °C (c = 0.03 mg/mL).

To obtain information on the chiroptical properties of the synthetic block copolymer, CD and UV-vis spectra were measured in THF at 25 °C (*c* = 0.03 mg/mL) (Figure 7). As shown in Figure 7, poly(**1**_25_-*b*-**3**_60_) and poly(**1**_25_-*b*-**4**_60_) had the same absorption profiles, with the maximum located at 364 nm. Poly(**1**_25_-*b*-**3**_60_) and poly(**1**_25_-*b*-**4**_60_) exhibited weak Cotton effects at 308 nm, indicating that the content of poly-**1**_25_ was low after polymerization. Poly(**1**_25_-*b*-**3**_60_) showed a negative Cotton effect at 364 nm, whereas poly(**1**_25_-*b*-**4**_60_) showed a positive Cotton effect at this absorption region. These results indicate that a helical conformation along the main chain with a preferred handedness of the polymers was formed, probably through the interaction of the polyisocyanide skeleton and chiral amide pedants.

**Figure 8:**
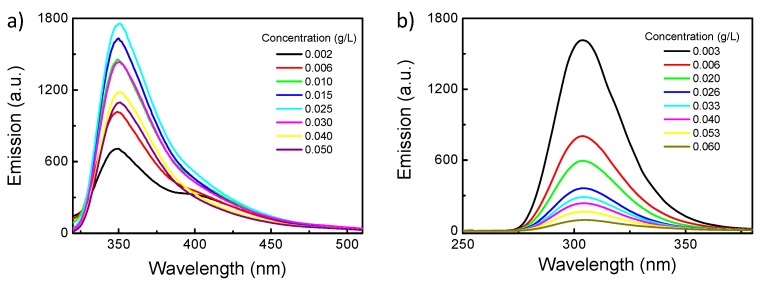
Emission spectra of poly-**1**_25_ (**a**) and poly(**1**_25_-*b*-**3**_30_) (**b**) in THF at different concentrations measured at room temperature (λexc(a) = 300 nm, λexc(b) = 210 nm).

During the investigation, fluorescence emission was observed from the model compounds poly-**1**_25_ and poly(**1**_25_-*b*-**3**_30_). As shown in Figure 8a, when the concentration increased from 0.001 to 0.025 g/L, the emission intensity at λ_em_ = 350 nm gradually increased. As shown in Figure 8b, the sample poly(**1**_25_-*b*-**3**_30_) was strongly emissive in the fluorescence spectra at a concentration of 0.003 g/L in THF. When the concentration increased from 0.003 to 0.06 g/L, the emission intensity at λ_em_ = 304 nm gradually decreased. Unexpectedly, the emission intensity dropped to 5% of that the highest intensity (0.003 g/L) as the concentration increased from 0.003 to 0.06 g/L. The polymer underwent aggregation-caused quenching (ACQ) effect in the aggregated state.

**Figure 9:**
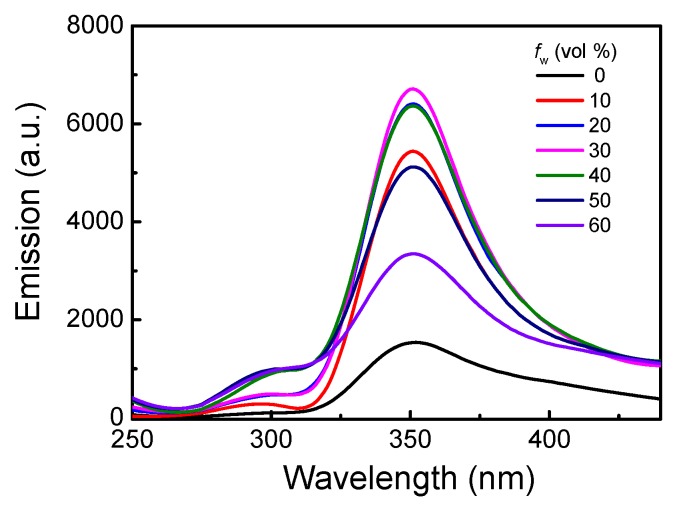
Emission spectra of poly(**1**_25_-*b*-**3**_30_) in THF/water mixtures with different water fractions (f_w_) at the concentration of 0.03 g/L (λexc = 230 nm).

The fluorescence behavior of poly(**1**_25_-*b*-**3**_30_) was systematically studied in THF/water mixtures with different water volume fractions (f_w_) at a concentration of 0.03 g/L, based on the consideration that water is a poor solvent for these compounds and may induce molecular aggregation. The fluorescence spectra of poly(**1**_25_-*b*-**3**_30_) are shown in Figure 9. In a pure THF solution, a weak emission peak was observed at 350 nm. As the water fraction increased from 10% to 30%, the emission gradually intensified. The strongest fluorescence intensity was observed at a f_w_ of 30%, with an intensity enhancement of approximately 5.6-fold compared to that of the pure THF solution. When the water content was greater than 30%, the emission gradually decreased.

**Figure 10:**
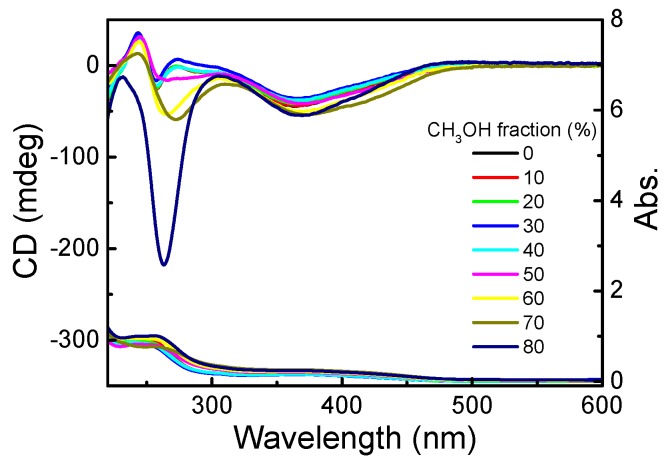
CD and UV-vis spectra of poly(**1**_25_-*b*-**3**_30_) measured in THF/CH_3_OH mixtures with different CH_3_OH fractions at the concentration of 0.03 g/L at room temperature.

To obtain the self-assembly structure, a polar protic solvent, CH_3_OH, was added to the THF solution of the poly(**1**_25_-*b*-**3**_30_) at room temperature. Figure 10 shows the CD and UV-vis spectra of poly(**1**_25_-*b*-**3**_30_) in different THF/CH_3_OH mixtures. Interestingly, the CD spectra of the copolymer showed distinct Cotton effects at 270 nm upon addition of CH_3_OH. As shown in Figure 10, the copolymers showed a weak Cotton effect at 270 nm in THF. For 0–40% CH_3_OH in THF, there was no change in the Cotton value of the copolymer at 270 nm. For a CH_3_OH fraction of 50%, the CD signal intensity at 270 nm showed some changes. By further increasing the CH_3_OH amount to 70%, the CD signals at 270 nm increased. When the volume of CH_3_OH was equal to 80%, the CD signals intensity at 270 nm dramatically increased. Thus, the Cotton effects of the poly(**1**_25_-*b*-**3**_30_) at approximately 270 nm in THF/ CH_3_OH were assigned to the benzene ring skeleton. These results suggested that block copolymers may self-assemble into achiral aggregates.

**Figure 11:**
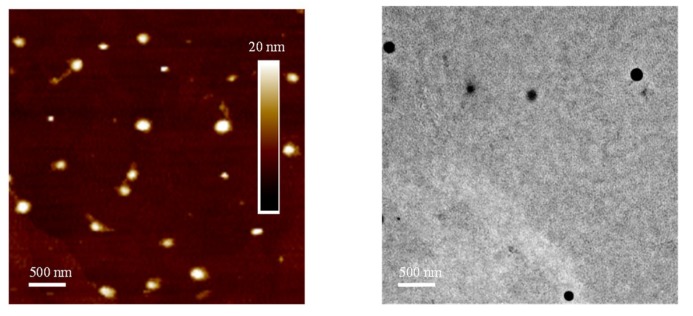
AFM height image (**a**) and TEM image (**b**) of the self-assembled structures of poly(**1**_25_-*b*-**3**_30_) in THF/CH_3_OH solutions (*v*:*v* = 2:8).

The self-assembly morphology of the aggregates was then investigated using atomic force microscopy (AFM) by spin-casting from the THF/CH_3_OH (*v*:*v* = 2:8) solution of poly(**1**_25_-*b*-**3**_30_) at room temperature (c = 0.20 g/L). As shown in Figure 11a, well-defined spherical nanoparticles were clearly observed in the taping mode AFM image. The average diameter of the particles was estimated to be approximately 105 nm. The self-assembled structures of poly(**1**_25_-*b*-**3**_30_) in THF/CH_3_OH solutions (*v*:*v* = 2:8) were further studied by transmission electron microscopy (TEM). As shown in Figure 11b, the TEM image confirmed the formation of homogeneous nanoparticles, and the diameters were determined to be approximately 110 nm. The poly(**1**_25_-*b*-**3**_30_) was able to self-assemble into spherical nanostructures in the selective THF/CH_3_OH solution.

## 4. Conclusions

*m*-Phenylene ethynylene bearing an amide linkage pendant was synthesized and polymerized to form a novel predominantly one-handed helical structure *m*-PPE. Poly-**1**_25_ showed a positive CD signal at a wavelength of approximately 308 nm and a negative second Cotton effect at a wavelength of approximately 289 nm in acetonitrile, THF, 1,4-dioxane, and H_2_O. Poly-**1**_25_ underwent a unique conformational change, such as a helix–helix transition of the polymer backbone, in mixed solvents without the assistance of extraneous molecules. The helical sense of poly-**1**_25_ was able to be controlled by changing the solvent composition. The polymer had a negative CD signal at a wavelength of approximately 308 nm and a positive CD signal at a wavelength of approximately 289 nm in the THF/CHCl_3_ mixed solvent, and the signs of these CD signals were opposite to those in pure THF at the same concentration, indicating that the polymer assumed helical conformations with reversed senses in the mixture solvents. We concluded that optically active poly(*m*-phenylacetylenes) underwent a unique helix–helix transition as a response to a mixed solvent stimuli. Finally, amphiphilic poly(meta-phenylene ethynylene)-*block*-polyisocyanide block copolymers containing hydrophilic PPE and hydrophobic PPI segments were facilely prepared using a one-pot synthesis method. These block copolymers can self-assemble into well-defined spherical nanostructures in selective solvents. We believe that the present study will provide a facile one-pot synthetic methodology for the preparation of functional optically active (co)polymers, which can be used as semiconducting and chiral materials in the future.

## Figures and Tables

**Table 1:** Results for the polymerization of monomer **1** using the Pd(II) Initiator in THF at 55 °C *^a^*.

Run	Polymer *^b^*	[**1**]0/[Pd]0 *^c^*	*M*_n_(kDa) *^d^*	*M*_w_/*M*_n_*^d^*	Yield *^e^* (%)
1	poly-**1**_10_	10	2.51	1.20	67
2	poly-**1**_15_	15	3.74	1.19	67
3	poly-**1**_20_	20	5.07	1.21	70
4	poly-**1**_25_	25	6.38	1.17	70
5	poly-**1**_30_	30	7.42	1.18	73
6	poly-**1**_35_	35	8.57	1.21	78

*^a^* The polymers were prepared according to the Scheme 1 in main text. *^b^* The footnote indicates the initial feed ratio of monomer **1** to the Pd(II) initiator. *^c^* The initial feed ratio of monomer **1** to the Pd(II) initiator. *^d^ M*_n_ and *M*_w_/*M*_n_ values were measured by SEC using polystyrene standards. *^e^* The isolated yield.

**Table 2:** Results for the One-Pot Synthesis of PPE-*b*-PPI Block Copolymers ^a^.

Run	Block Polymer *^b^*	*M*_n_ (kDa) *^c^*	*M*w/*M*_n_*^c^*	Yield *^d^* (%)
1	poly(**1**_25_-*b*-**2**_15_)	9.08	1.15	73
2	poly(**1**_25_-*b*-**2**_50_)	17.80	1.21	78
3	poly(**1**_25_-*b*-**2**_100_)	31.75	1.17	81
4	poly(**1**_25_-*b*-**3**_20_)	11.64	1.14	78
5	poly(**1**_25_-*b*-**3**_60_)	25.33	1.09	81
6	poly(**1**_25_-*b*-**4**_20_)	11.16	1.16	77
7	poly(**1**_25_-*b*-**4**_30_)	14.15	1.15	81
8	poly(**1**_25_-*b*-**4**_60_)	22.86	1.21	80

*^a^* The block copolymers were synthesized according to Scheme 1 by first preparing the macroinitiator, followed by the addition of monomer **2**, **3**, or **4** as denoted. *^b^* The footnotes indicate the initial feed ratios of monomer to initiator ([M]0/[Pd]0). *^c^* The Mn and Mw/Mn values were determined by SEC and reported as their polystyrene equivalents. *^d^* Isolated yield over the two steps.

## Supplementary Materials

The following are available online at http://www.mdpi.com/2073-4360/10/9/936/s1.
