# Supplementary material for: A Facile Synthetic Route to Amphiphilic Poly(Meta-Phenylene Ethynylene) and Poly(Meta-Phenylene Ethynylene)-Block-Polyisocyanide Using a Single Catalyst
Source: Polymers (Basel). 2018 Aug 23;10(9):936. doi: 10.3390/polym10090936 (PMC6403950; doi:10.3390/polym10090936)
Supplement: Supplementary file 1 [file polymers-10-00936-s001.pdf]

polymers

Supplementary Materials: A Facile Synthetic Route to Amphiphilic Poly(*meta*- phenylene ethynylene) and Poly(*meta*-phenylene ethynylene)-*block*-Polyisocyanide Using a Single Catalyst

Chonglong Li, Xunhui Xu, Lei Xu and Na Liu

1. Synthetic monomer procedures

Synthesis of monomer 1

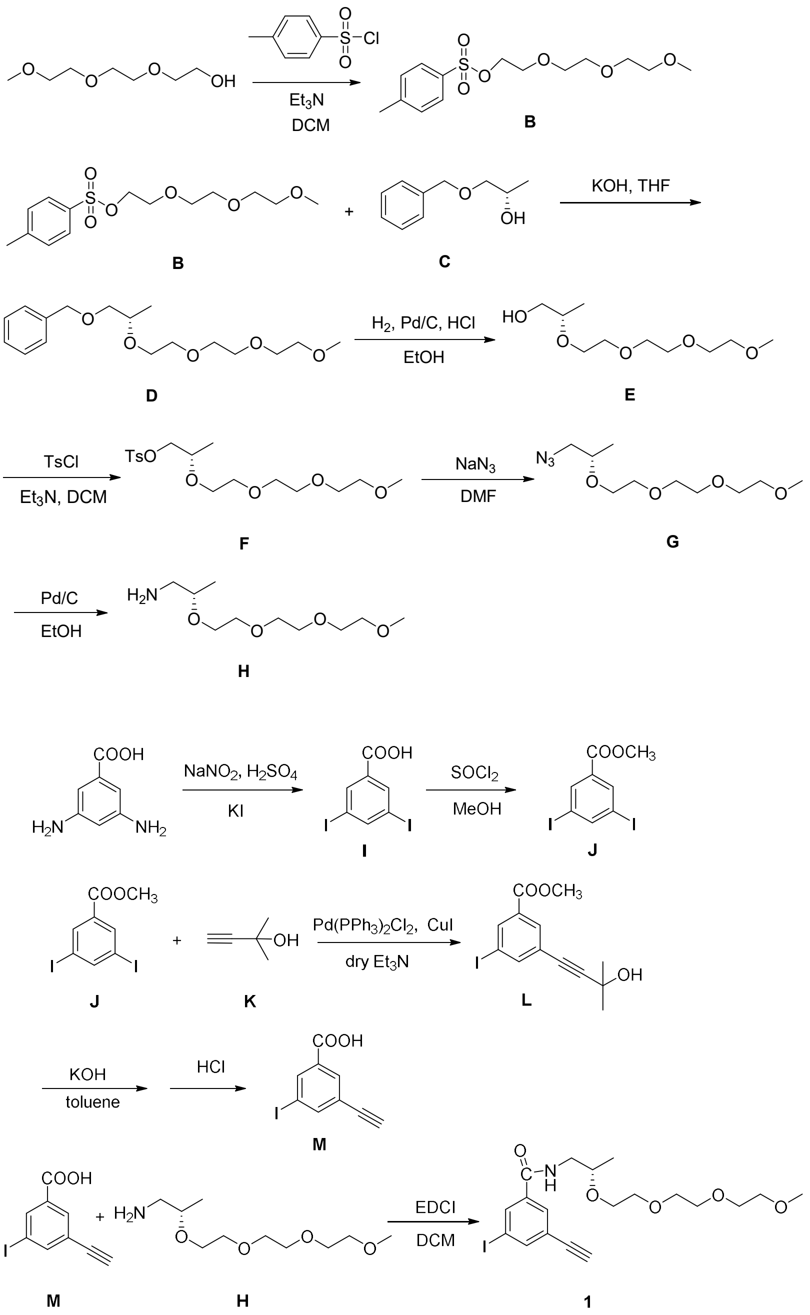

Scheme S1. Synthesis of monomer **1**.

Synthesis of compound B

Triethyleneglycol monomethyl ether (32.8 g, 200 mmol) was dissolved in CH_2Cl_2 (100 mL) and triethylamine (83 mL). The resulting mixture was cooled to 0 °C and a solution of 4-toluenesulfonyl chloride (41.9 g, 220 mmol) in CH_2Cl_2 (220 mL) was added drop-wise at 0 °C. After the reaction mixture was stirred at room temperature for 12 h, white precipitate was filtrated off and the residue was washed with ethyl acetate. The organic layer was washed with water and brine, passed through anhydrous Na_2SO_4 and concentrated. The crude product was purified by column chromatography (hexane: ethylacetate = 1: 1) to obtain viscous colorless liquid compound **B** (53.0 g, 83.3%).

^{1}H NMR (600 MHz, $CDCl_3$, 25 °C) δ: 7.78 (d, *J* = 8.3 Hz, 1H), 7.33 (d, *J* = 8.3 Hz, 1H), 4.15 (t, *J* = 4.8 Hz, 2H), 3.68–3.51 (m, 10H), 3.35 (s, 3H), 2.43 (s, 3H).

Synthesis of compound D

A solution of compound B (33.0 g, 103 mmol), compound C (15.0 g, 90 mmol) and KOH powder (21.2 g, 378 mmol) were taken together in a round bottom flask along with 220 mL of dry THF. The reaction mixture was stirred at 80 °C for 36 h under nitrogen atmosphere, during which time lots of white solid came out of the reaction mixture. When the reaction was over, the reaction mixture was allowed to cool to room temperature and poured into cold water (120 mL) carefully. The mixture was stirred for 1 h and extracted with CH_2Cl_2. The organic layer was dried over anhydrous Na_2SO_4, filtered, and concentrated. The crude product was purified by column chromatography (silica, ethyl acetate/hexane, 1:1) to get a colourless oil product compound D (20.7 g, 73.6%).

^{1}H NMR (600 MHz, $CDCl_3$, 25 °C) δ: 7.33(m, 5H), 4.54 (d, *J* = 5.5 Hz, 2H), 3.69-3.39 (m, 15H), 3.36 (s, 3H), 1.17 (d, *J* = 6.3 Hz, 3H).

Synthesis of compound E

Compound D (17.2 g, 55 mmol) was dissolved in ethanol (250 mL) and acidified with HCl (0.1 mL, 37%). A catalytic amount of Pd/C (10%, 1.72 g) was added to the reaction solution. The reaction mixture was then stirred at room temperature for 24 h under 1 atm of hydrogen. After the reaction was completed, the reaction solution was filtered and the ethanol was removed to obtain colourless oil compound **E** (12.0 g, 98%).

^{1}H NMR (600 MHz, $CDCl_3$, 25 °C) δ: 3.74–3.41 (m, 15H), 3.34(s, 3H), 3.00 (s, 1H), 1.07 (d, *J* = 6.3 Hz, 2H).

Synthesis of compound F

Compound **E** (11.5 g, 51.7 mmol) was dissolved in CH_2Cl_2 (50 mL) and triethylamine (22 mL). The resulting mixture was cooled to 0 °C and a solution of 4-toluenesulfonyl chloride (12.1 g, 63.4 mmol) in CH_2Cl_2 (200 mL) was added drop-wise at 0 °C over 0.5 h. After the reaction mixture was stirred at room temperature for 12 h, white precipitate was filtrated off and the residue was washed with ethyl acetate. The organic layer was washed with water and brine, dried over anhydrous Na_2SO_4, filtered, and concentrated. The crude product was purified by column chromatography (hexane: ethylacetate = 1: 1) to obtain colorless liquid compound **F** (16.0 g, 83%).

^{1}H NMR (600 MHz, $CDCl_3$, 25 °C) δ: 7.78 (d, *J* = 8.2 Hz, 2H), 7.33 (d, *J* = 8.2 Hz, 2H), 3.99-3.87 (m, 2H), 3.69–3.52 (m, 13H), 3.35 (s, 3H), 2.42 (s, 3H), 1.11 (d, *J* = 6.4, 3H). ^{13}C NMR (150 MHz, $CDCl_3$) δ: 144.91, 132.95, 129.93, 128.04, 73.55, 72.70, 71.99, 70.76, 70.67, 70.59, 68.96, 59.13, 21.76, 16.85. FT-IR (KBr, cm^{-1}): 1094 (ν_{C-O-C}). MS m/z calcd for $C_{17}H_{28}NaO_7S$ $[M + Na]^+$: 399.1453; Found: 399.1466. Anal. Calcd (%) for $C_{17}H_{28}O_7S$: C, 54.24; H, 7.50. Found: C, 54.27%; H, 7.51%.

Synthesis of compound G

Compound **F** (8.1 g, 21.5 mmol) was dissolved in dry DMF (160 mL), NaN_3 (7.0 g, 107.0 mmol) was added to the reaction bottle. The reaction mixture was stirred at 100 °C for 12 h. After the reaction was completed, 50 mL of water was added to the reaction mixture and concentrated. 50 mL of water was added to the concentrated mixture and extracted in DCM. The organic layer was dried over

anhydrous Na_2SO_4, filtered, and concentrated. The crude product was purified by column chromatography (hexane: ethylacetate = 1: 1) to get the liquid compound G (5.2 g, 97%).

^{1}H NMR (600 MHz, $CDCl_3$, 25 °C) δ: 3.74-3.69 (m, 1H), 3.68–3.58 (m, 10H), 3.55–3.51 (m, 2H), 3.36 (s, 3H), 3.25 (dd, *J* = 12.8, 6.8 Hz, 1H), 3.14 (dd, *J* = 12.8, 6.8 Hz, 1H), 1.17 (d, *J* = 6.3 Hz, 3H). ^{13}C NMR (150 MHz, $CDCl_3$) δ: 75.48, 72.04, 70.89, 70.76, 70.72, 70.61, 68.65, 59.10, 55.73, 17.61. FT-IR (KBr, cm^{-1}): 2095 (ν_{N3}). MS m/z calcd for $C_{10}H_{21}N_3NaO_4$ [M + Na]$^+$: 270.1430; Found: 270.1347. Anal. Calcd (%) for $C_{10}H_{21}N_3O_4$: C, 48.57; H, 8.56; N, 16.99. Found: C, 48.55%; H, 8.55%; N, 16.97%.

Synthesis of compound H

Compound **G** (5.0 g, 20.2 mmol) was dissolved in ethanol (150 mL). A catalytic amount of 10% Pd/C (0.5 g) was added to the solution. The reaction mixture was then stirred at room temperature for 24 h under 1 atm of hydrogen. After the reaction was completed, the reaction solution was filtered and the ethanol was removed to obtain colourless oil compound **H** (4.3 g, 96%).

^{1}H NMR (600 MHz, $CDCl_3$, 25 °C) δ: 3.78–3.43(m, 13H), 3.36 (s, 3H), 2.79–2.50 (m, 2H), 2.14 (m, 2H), 1.11 (d, *J* = 6.3 Hz, 3H). ^{13}C NMR (150 MHz, $CDCl_3$, 25 °C) δ: 77.71, 72.02, 70.94, 70.71, 70.66, 70.61, 68.04, 59.12, 47.58, 17.35. FT-IR (KBr, cm^{-1}): 2870 (ν_{C-H}), 1092 (ν_{C-O-C}). MS m/z calcd for $C_{10}H_{24}NO_4$ [M + H]$^+$: 222.1705; Found: 222.1964. Anal. Calcd (%) for $C_{10}H_{23}NO_4$: C, 54.28; H, 10.48; N, 6.33. Found: C, 54.25%; H, 10.49%; N, 6.32%.

Synthesis of compound I

An aqueous solution of sodium nitrite (2.50 M, 136 mL, 0.34 mol) was added to a dispersion solution of 3, 5-diaminobenzoic acid (24.0 g, 0.16 mol) in 25% sulfuric acid (560 mL) at 0 °C. After stirred at 0 °C for 2 h, urea (24.0 g, 0.40 mol) was added. 120 mL aqueous solution of potassium iodide (131.2 g, 0.79 mol) was then added dropwise to the reaction mixture at 0 °C over 2 h. After being stirred at ambient temperature overnight, the brown solid was collected by filtration, washed with water, and dried under vacuum. The crude product was purified by column chromatography (CH_2Cl_2/MeOH = 50/1) to get 3, 5-diiodobenzoic acid (12.94 g, 21.2%).

^{1}H NMR (600 MHz, DMSO–d_6, 25 °C) δ: 13.51 (broad s, 1H), 8.33 (s, 1H), 8.17 (s, 2H).

Synthesis of compound J

To a stirred solution of compound I (12.0 g, 32.2 mmol) in MeOH (240 mL), $SOCl_2$ (12.0 mL, 7.8 eq.) was added slowly at 0 °C. After the reaction was stirred at 80 °C for 12 h, the reaction mixture was allowed to cool to room temperature and concentrated under reduced pressure. The crude product was purified by column chromatography to afford white solid compound **J** (12.0 g, 96%).

^{1}H NMR (600 MHz, $CDCl_3$, 25 °C) δ: 8.32 (s, 2H), 8.22 (s, 1H), 3.92 (s, 3H).

Synthesis of compound L

A suspension of compound **J** (9.38 g, 24.2 mmol), $Pd(PPh_3)_2Cl_2$ (170 mg, 0.24 mmol), copper (I) iodide (88 mg, 0.46 mmol) was degassed for three times. Triethylamine (160 mL) was added under N_2, and then 2-Methyl-3-butyn-2-ol (2.20 g, 26.6 mmol) was added drop-wise. After the reaction mixture was stirred at 80 °C for 14 h, the solvent was removed by evaporation under reduced pressure. The residue was dissolved in CH_2Cl_2, washed with saturated $NaHCO_3$, water, and brine. The organic layer was dried over Na_2SO_4, filtered, and concentrated. The crude product was purified by column chromatography (CH_2Cl_2: PE = 5: 1) to afford compound **L** as a colorless oil (4.24 g, 51%).

^{1}H NMR (600 MHz, $CDCl_3$, 25 °C) δ: 8.28 (s, 1H), 8.01 (s, 1H), 7.91 (s, 1H), 3.91 (s, 3H), 2.21 (s, 1H), 1.60 (s, 6H). ^{13}C NMR (150 MHz, $CDCl_3$, 25 °C) δ: 165.10, 144.20, 138.15, 132.07, 131.86, 125.14, 96.22, 93.29, 79.73, 65.66, 52.71, 31.44. FT-IR (KBr, cm^{-1}): 3387 (ν_{O-H}), 2980 (ν_{C-H}), 1725 ($\nu_{C=O}$). MS m/z calcd for $C_{13}H_{12}IO_2$ [M–OH]$^+$: 326.9882; Found: 326.9958. Anal. Calcd (%) for $C_{13}H_{13}IO_3$: C, 45.37; H, 3.81. Found: C, 45.35%; H, 3.80%.

Synthesis of compound M

Under an atmosphere of nitrogen, KOH (1.03 g, 18.4 mmol) was added to a solution of compound L (2.8 g, 9.4 mmol) in toluene (100 mL). After the reaction mixture was stirred at 25 °C for 10 h, and then at 80 °C for 10 h, the reaction mixture was poured into cold water and extracted with CH_2Cl_2 for three times. The water phase was adjusted pH = 1 by using 3 M HCl and extracted with EtOAc (100 mL × 3). The combined organic phase was washed with H_2O (50 mL × 3) and brine (100 mL × 2), and then dried over anhydrous $MgSO_4$. After filtration, the solvent was removed by evaporation under reduced pressure and the crude product was purified by column chromatography (EA: PE = 5: 1) to afford white solid compound M (1.60 g, 59%).

^{1}H NMR (600 MHz, $(CD_3)_2SO$, 25 °C) δ: 13.47 (s, 1H), 8.22 (s, 1H), 8.07 (s, 1H), 7.92 (s, 1H), 4.42 (s, 1H). ^{13}C NMR (150 MHz, $(CD_3)_2SO$, 25 °C) δ: 165.10, 143.44, 138.02, 133.00, 131.49, 124.03, 94.79, 83.07, 80.90. FT-IR (KBr, cm^{-1}): 1684 ($\nu_{C=O}$). MS m/z calcd for $C_9H_4IO_2$ [M–H]$^+$: 270.9256; Found: 270.9279. Anal. Calcd (%) for $C_9H_5IO_2$: C, 39.74; H, 1.85. Found: C, 39.77%; H, 1.84%.

Synthesis of compound 1

To a 250-mL round-bottomed flask containing a magnetic stirring bar were added compound **M** (1.50 g, 5.50 mmol) and EDC·HCl (1.24 g, 6.5 mmol) in CH_2Cl_2 (60 mL) under N_2. A solution of compound **H** (1.11 g, 5.0 mmol) in dry CH_2Cl_2 (2.0 mL) was added at 0 °C. The mixture was stirred at 0 °C for 15 min, and then at room temperature for 2 h. The reaction mixture was treated with 2 M HCl aqueous solution and saturated NH_4Cl aqueous solution, and the organic layer was separated. The aqueous layer was extracted with CH_2Cl_2. Then, the combined organic layers were dried over Na_2SO_4, filtered and concentrated *in vacuo*. The crude product was purified by column chromatography on silica gel (eluent: hexane/EtOAc = 1: 2) to afford compound **1** as a colorless oil (1.50 g, 58%).

^{1}H NMR (600 MHz, $CDCl_3$, 25 °C) δ: 8.17 (s, 1H), 7.91 (s, 1H), 7.89 (s, 1H), 7.28 (s, 1H), 3.77-3.54 (m, 12H), 3.45 (m, 2H), 3.30(s, 3H), 3.26-3.19 (m, 1H), 3.16 (s, 1H), 1.19 (d, *J* = 6.2 Hz, 3H). ^{13}C NMR (150 MHz, $CDCl_3$, 25 °C) δ: 165.25, 143.11, 136.88, 136.81, 130.16, 124.36, 93.62, 81.39, 79.46, 74.89, 71.99, 70.95, 70.71, 70.57, 70.53, 68.14, 59.08, 45.31, 17.88. FT-IR (KBr, cm^{-1}): 3289 (ν_{N-H}), 1645 ($\nu_{C=O}$). MS m/z calcd for $C_{19}H_{26}INNaO_5$ [M + Na]$^+$: 498.0753; Found: 498.0783. Anal. Calcd (%) for $C_{19}H_{26}INO_5$: C, 48.01; H, 5.51; N, 2.95. Found: C, 48.05%; H, 5.52%; N, 2.94%.

2. Tables S1–S3

Table S1. Results for the one-pot synthesis of poly($\mathbf{1}_m$-*b*-$\mathbf{2}_n$) block copolymers[a].

Run	Polymer[b]	M_n[c] (kDa)	M_w/M_n[c]	Yield[d] (%)
1	poly($\mathbf{1}_{25}$-*b*-$\mathbf{2}_{15}$)	9.08	1.15	73
2	poly($\mathbf{1}_{25}$-*b*-$\mathbf{2}_{25}$)	11.89	1.15	75
3	poly($\mathbf{1}_{25}$-*b*-$\mathbf{2}_{35}$)	13.82	1.17	73
4	poly($\mathbf{1}_{25}$-*b*-$\mathbf{2}_{50}$)	17.80	1.21	78
5	poly($\mathbf{1}_{25}$-*b*-$\mathbf{2}_{60}$)	21.53	1.20	79
6	poly($\mathbf{1}_{25}$-*b*-$\mathbf{2}_{100}$)	31.75	1.17	81

[a]The block copolymers were synthesized according to Scheme 2 by first preparing the macroinitiator, followed by the addition of monomer **2** as denoted. [b]The footnotes indicate the initial feed ratios of monomer to initiator ($[M]_0/[Pd]_0$). [c]The M_n and M_w/M_n values were determined by SEC and reported as their polystyrene equivalents. [d]Isolated yield over the two steps.

Table S2. Results for the one-pot synthesis of poly(**1**$_m$-*b*-**3**$_n$) block copolymers[a].

Run	Polymer[b]	M_n[c] (kDa)	M_w/M_n[c]	Yield[d] (%)
1	poly(**1**$_{25}$-*b*-**3**$_{10}$)	8.40	1.16	73
2	poly(**1**$_{25}$-*b*-**3**$_{15}$)	10.25	1.14	76
3	poly(**1**$_{25}$-*b*-**3**$_{20}$)	11.64	1.14	78
4	poly(**1**$_{25}$-*b*-**3**$_{30}$)	13.75	1.10	76
5	poly(**1**$_{25}$-*b*-**3**$_{35}$)	15.68	1.14	80
6	poly(**1**$_{25}$-*b*-**3**$_{45}$)	18.88	1.15	83
7	poly(**1**$_{25}$-*b*-**3**$_{60}$)	25.33	1.09	81

[a]The block copolymers were synthesized according to Scheme 2 by first preparing the macroinitiator, followed by the addition of monomer **3** as denoted. [b]The footnotes indicate the initial feed ratios of monomer to initiator ($[M]_0/[Pd]_0$). [c]The M_n and M_w/M_n values were determined by SEC and reported as their polystyrene equivalents. [d]Isolated yield over the two steps.

Table S3. Results for the one-pot synthesis of poly(**1**$_m$-*b*-**4**$_n$) block copolymers[a].

Run	Polymer[b]	M_n[c] (kDa)	M_w/M_n[c]	Yield[d] (%)
1	poly(**1**$_{25}$-*b*-**4**$_{10}$)	8.08	1.12	71
2	poly(**1**$_{25}$-*b*-**4**$_{15}$)	9.78	1.14	74
3	poly(**1**$_{25}$-*b*-**4**$_{20}$)	11.16	1.16	77
4	poly(**1**$_{25}$-*b*-**4**$_{25}$)	12.50	1.16	80
5	poly(**1**$_{25}$-*b*-**4**$_{30}$)	14.15	1.15	81
6	poly(**1**$_{25}$-*b*-**4**$_{50}$)	18.89	1.18	82
7	poly(**1**$_{25}$-*b*-**4**$_{60}$)	22.86	1.21	80

[a]The block copolymers were synthesized according to Scheme 2 by first preparing the macroinitiator, followed by the addition of monomer **4** as denoted. [b]The footnotes indicate the initial feed ratios of monomer to initiator ($[M]_0/[Pd]_0$). [c]The M_n and M_w/M_n values were determined by SEC and reported as their polystyrene equivalents. [d]Isolated yield over the two steps.

3. Figures S1–S31

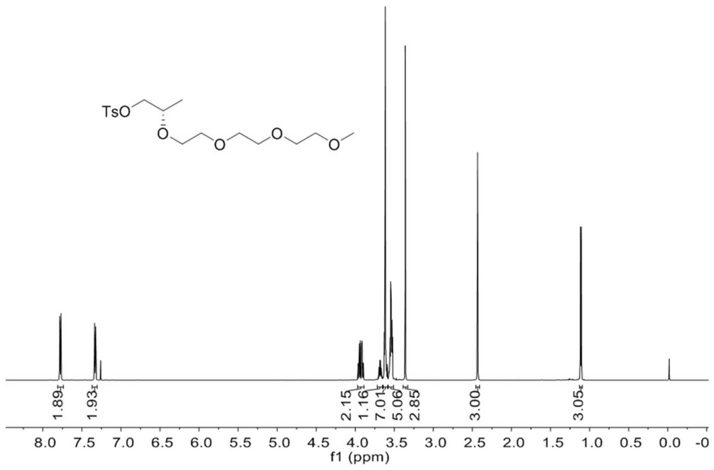

Figure S1. ^{1}H NMR (600 MHz) spectra of compound **F** measured in $CDCl_3$ at 25 °C.

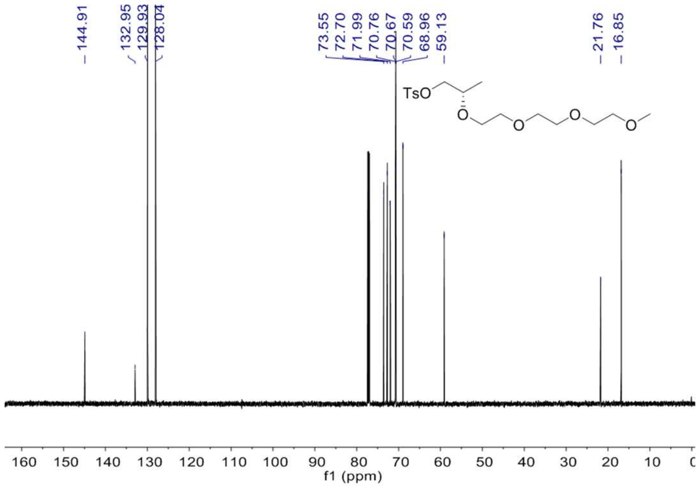

Figure S2. ^{13}C NMR (150 MHz) spectra of compound **F** measured in $CDCl_3$ at 25 °C.

Figure S3. FT-IR spectra of compound **F** measured at 25 °C using KBr pellets.

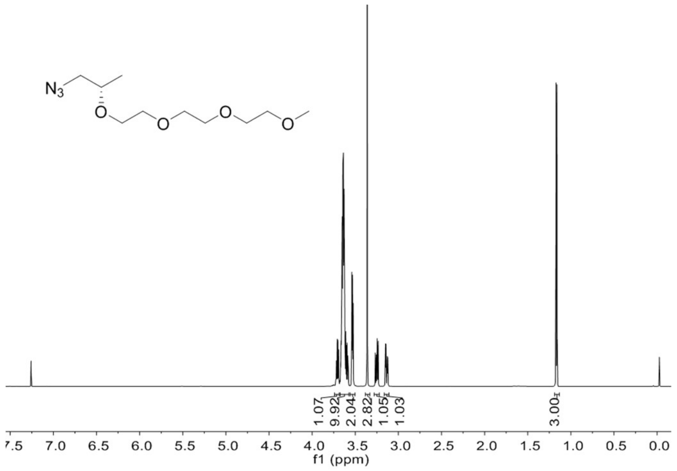

Figure S4. ^{1}H NMR (600 MHz) spectra of compound **G** measured in $CDCl_3$ at 25 °C.

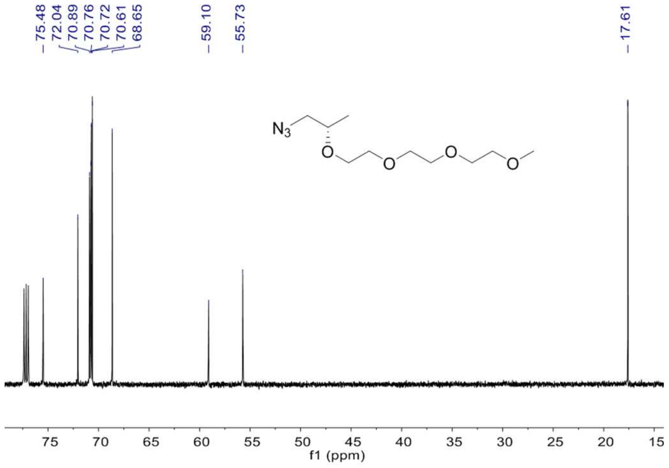

Figure S5. ^{13}C NMR (150 MHz) spectra of compound **G** measured in $CDCl_3$ at 25 °C.

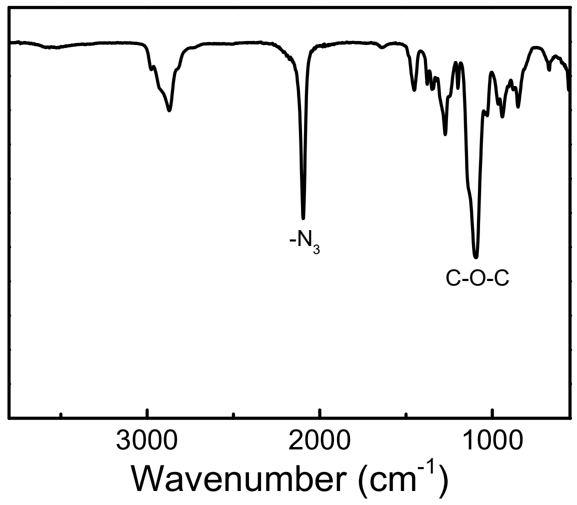

Figure S6. FT-IR spectra of compound **G** measured at 25 °C using KBr pellets.

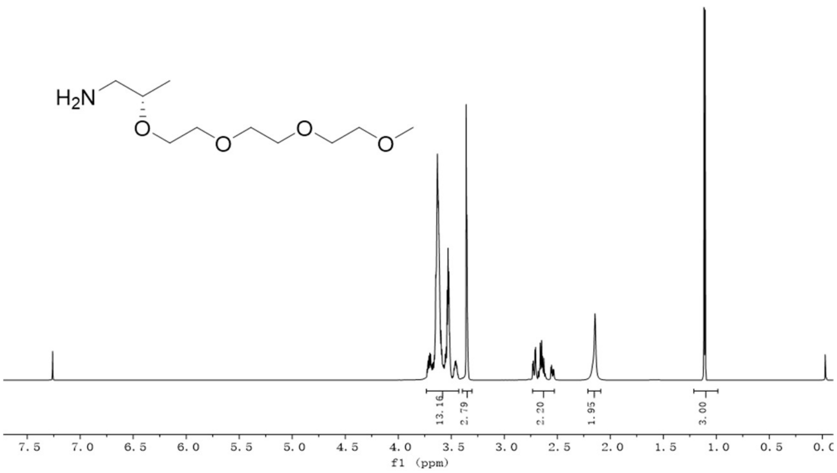

Figure S7. ^{1}H NMR (600 MHz) spectra of compound **H** measured in $CDCl_3$ at 25 °C.

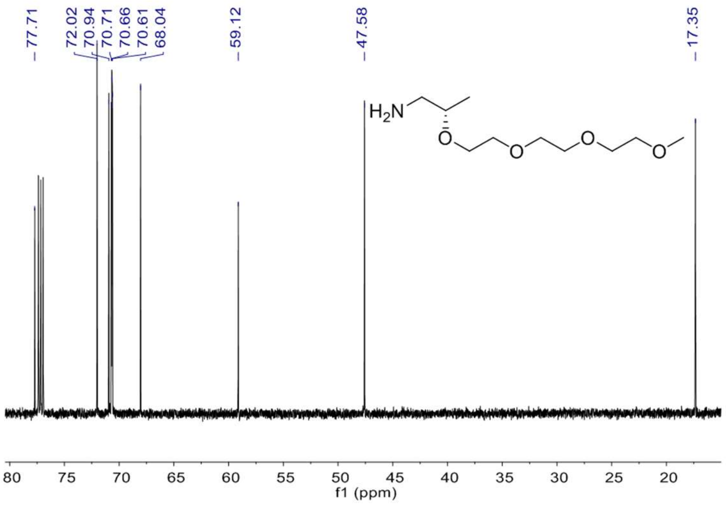

Figure S8. ^{13}C NMR (150 MHz) spectra of compound **H** measured in $CDCl_3$ at 25 °C.

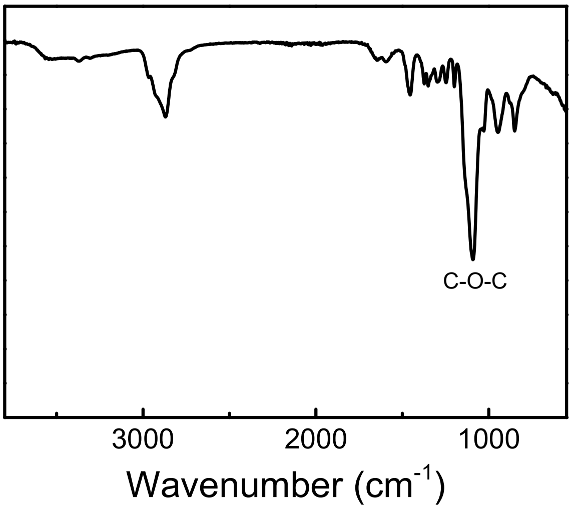

Figure S9. FT-IR spectra of compound **H** measured at 25 °C using KBr pellets.

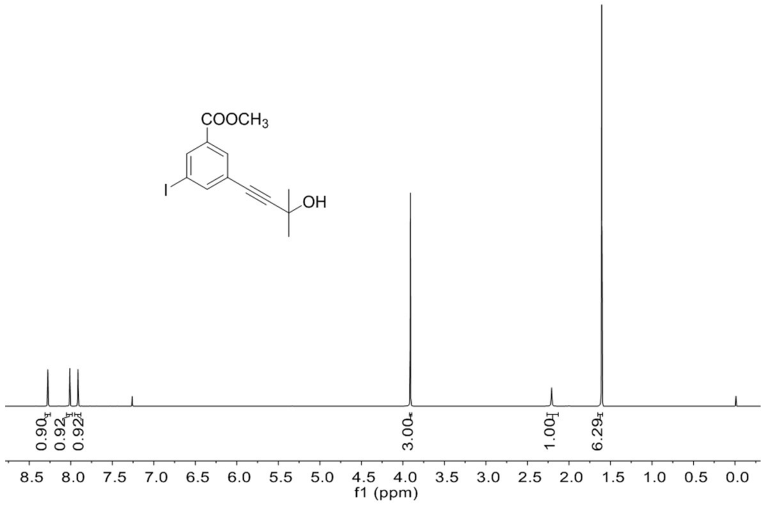

Figure S10. ^{1}H NMR (600 MHz) spectra of compound **L** measured in $CDCl_3$ at 25 °C.

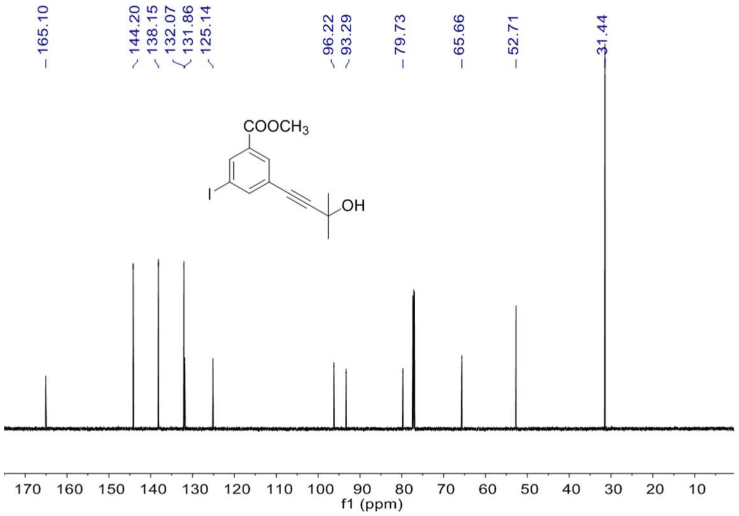

Figure S11. ^{13}C NMR (150 MHz) spectra of compound **L** measured in $CDCl_3$ at 25 °C.

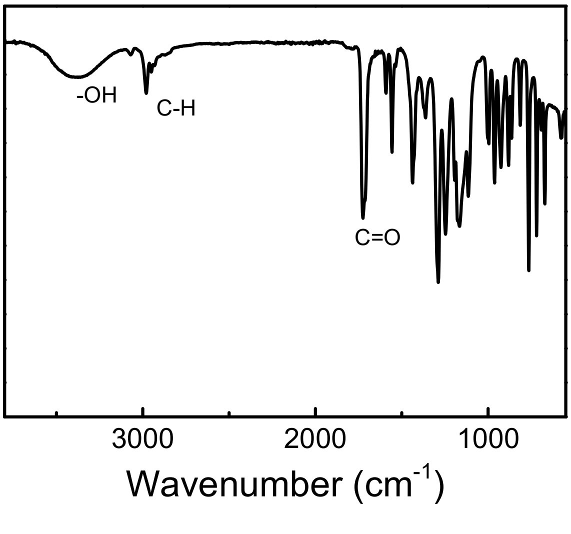

Figure S12. FT-IR spectra of compound **L** measured at 25 °C using KBr pellets.

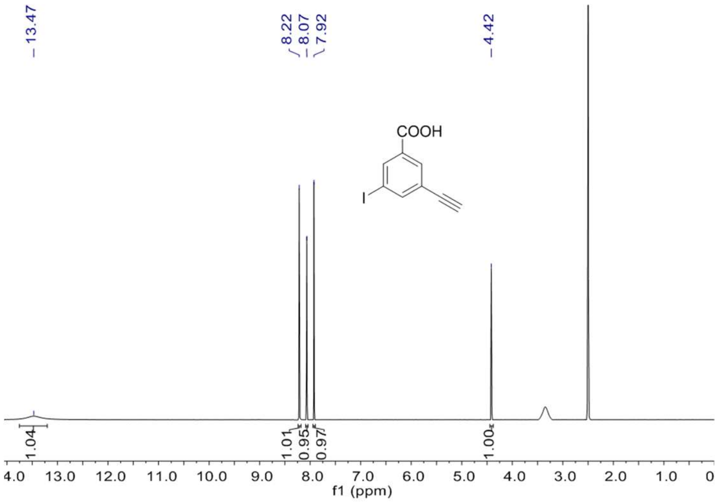

Figure S13. ^{1}H NMR (600 MHz) spectra of compound **M** measured in $(CD_3)_2SO$ at 25 °C.

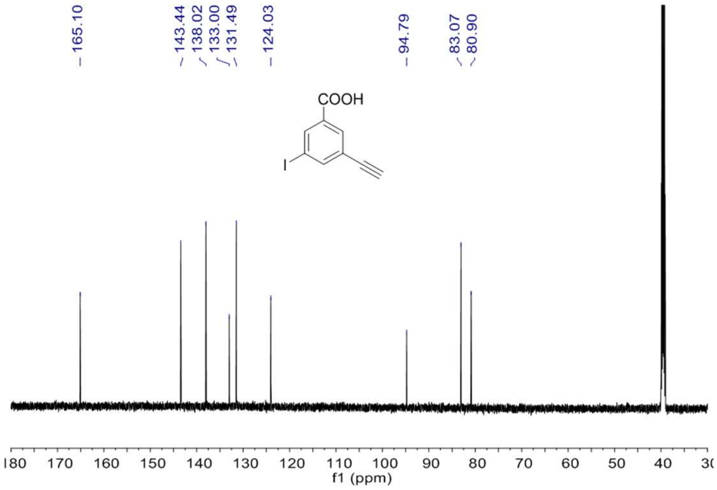

Figure S14. ^{13}C NMR (150 MHz) spectra of compound **M** measured in $(CD_3)_2SO$ at 25 °C.

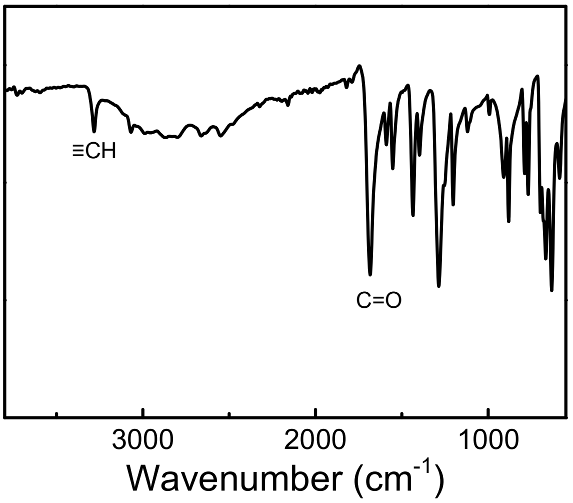

Figure S15. FT-IR spectra of compound **M** measured at 25 °C using KBr pellets.

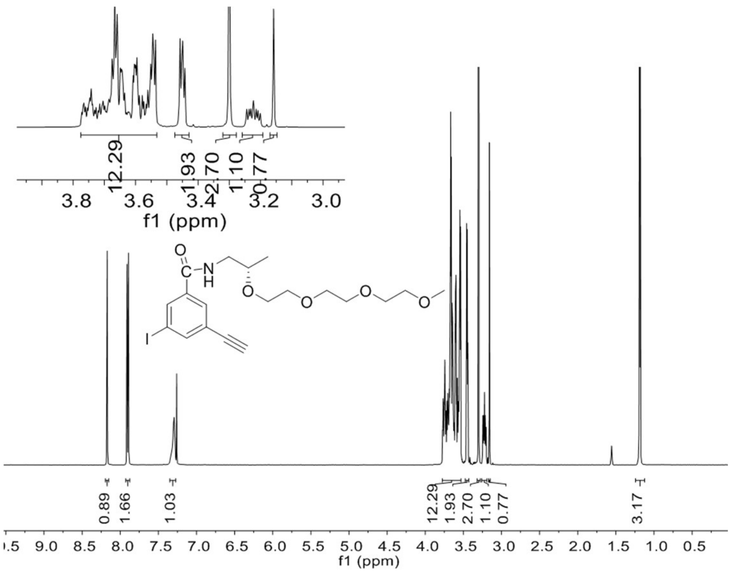

Figure S16. ^{1}H NMR (600 MHz) spectra of compound **1** measured in $CDCl_3$ at 25 °C.

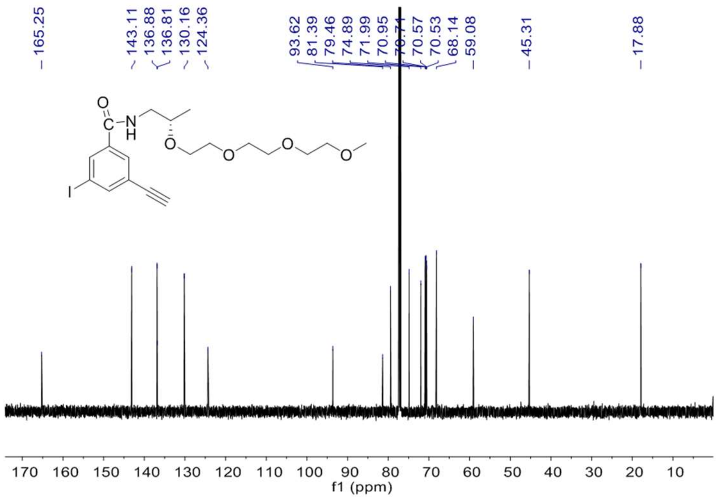

Figure S17. ^{13}C NMR (150 MHz) spectra of compound **1** measured in $CDCl_3$ at 25 °C.

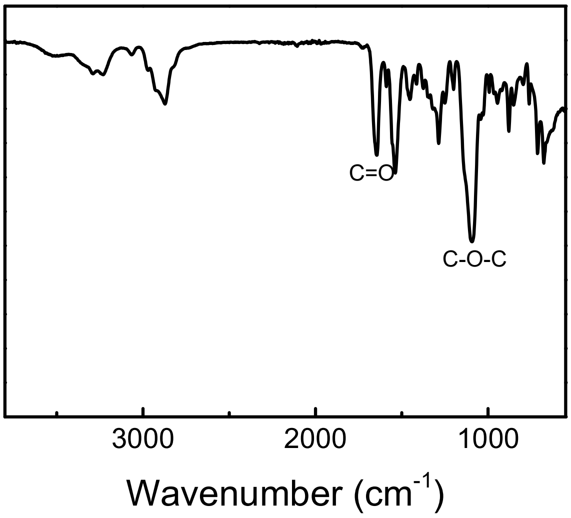

Figure S18. FT-IR spectra of compound **1** measured at 25 °C using KBr pellets.

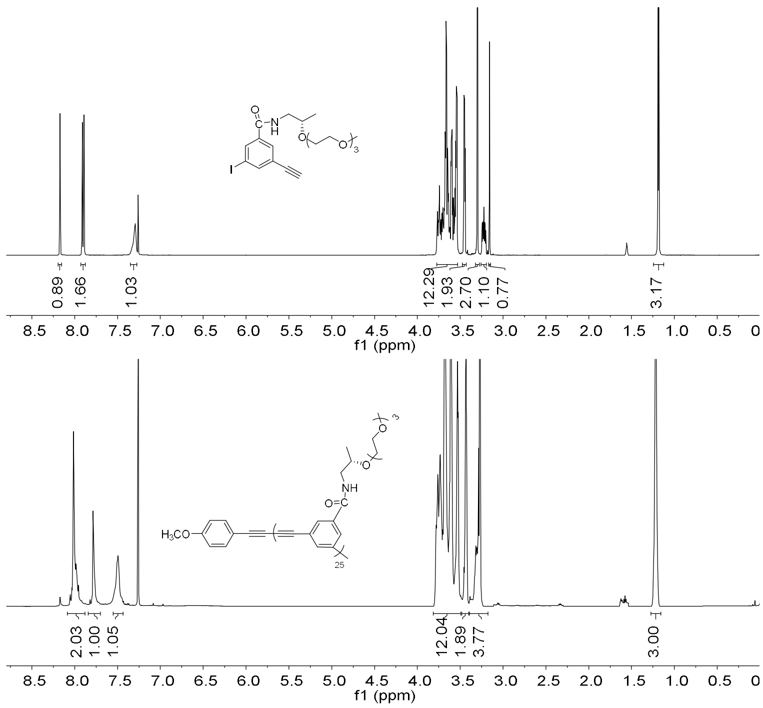

Figure S19. ^{1}H NMR (600 MHz) spectra of poly-$\mathbf{1}_{25}$ measured in $CDCl_3$ at 25 °C.

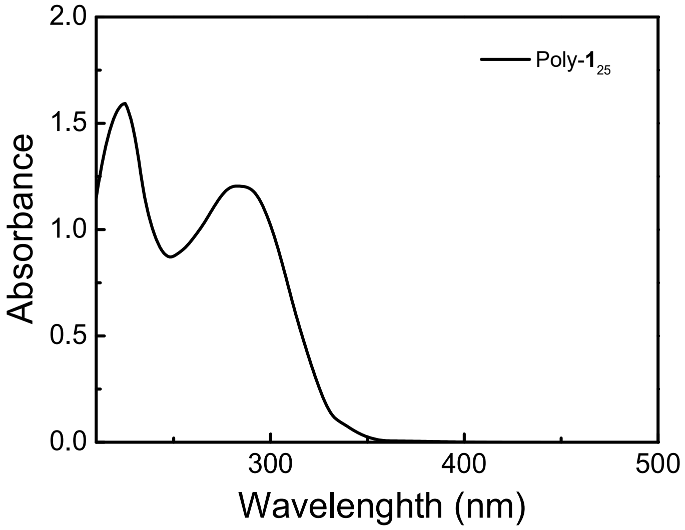

Figure S20. UV-vis absorption spectra of poly-$\mathbf{1}_{25}$ measured in THF at 25 °C (c = 0.03 g/L).

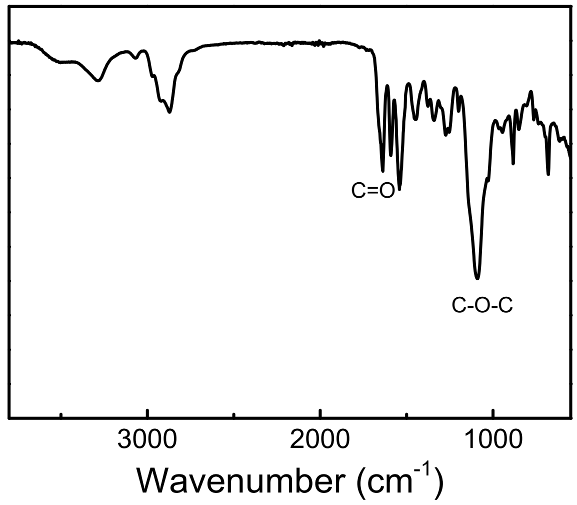

Figure S21. FT-IR spectra of poly-**1**25 measured at 25 °C using KBr pellets.

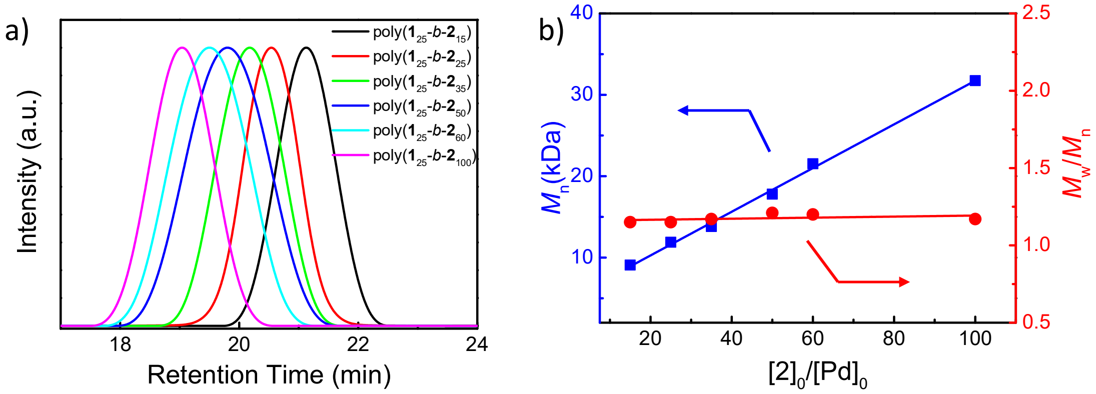

Figure S22. (**a**) SEC chromatograms of poly(**1**25-*b*-**2**n) prepared from the copolymerization of monomer **2** with the Pd(II)-terminated poly-**1**25 as macroinitiator in THF with different initial feed ratios of monomer to catalyst. (**b**) Plots of M_n and M_w/M_n values of poly(**1**m-*b*-**2**n)s measured as a function of the initial feed ratios of monomer **2** to the common macroinitiator, Pd(II)-terminated poly-**1**25 (M_n = 6.38 kDa, M_w/M_n = 1.17). SEC conditions: temperature = 40 °C; eluent = THF.

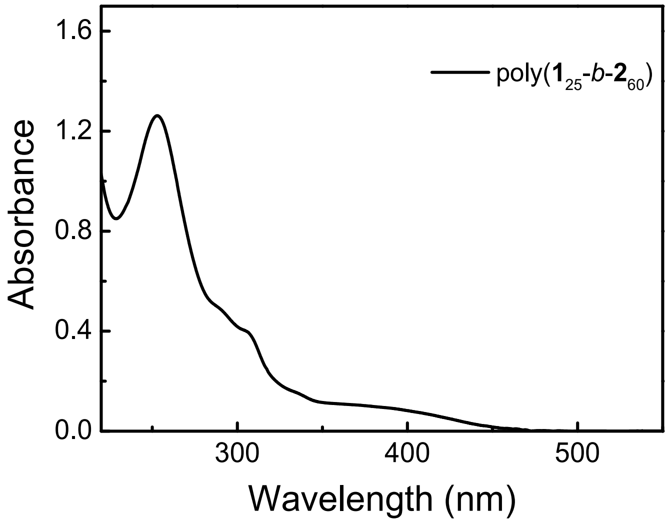

Figure S23. UV-vis absorption spectra of poly(**1**25-*b*-**2**60) measured in THF at 25 °C (*c* = 0.03 g/L).

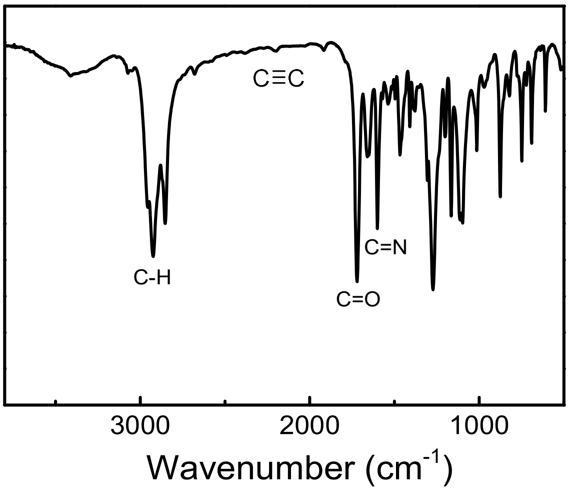

Figure S24. FT-IR spectra of poly($\mathbf{1}_{25}$-*b*-$\mathbf{2}_{60}$) measured at 25 °C using KBr pellets.

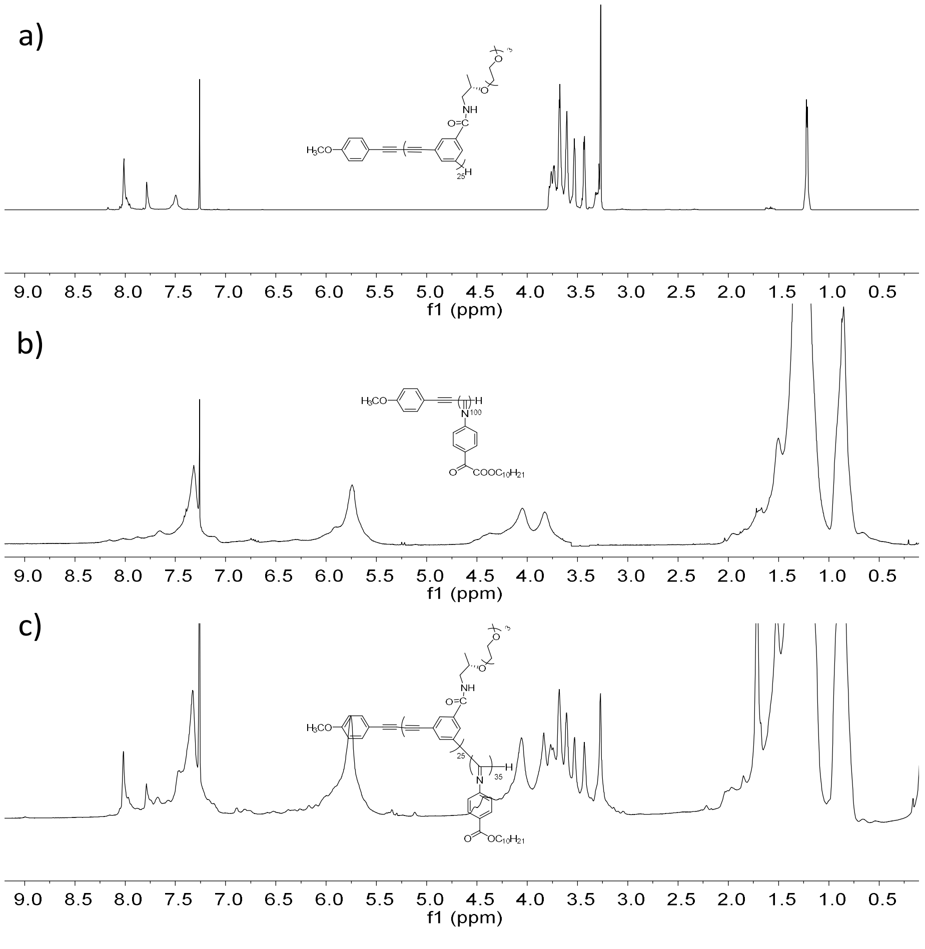

Figure 25. ^{1}H NMR spectra (600 MHz) of PPE homopolymer poly-$\mathbf{1}_{25}$ (**a**), PPI homopolymer poly-$\mathbf{2}_{100}$ (**b**), PPE-*b*-PPI block copolymer poly($\mathbf{1}_{25}$-*b*-$\mathbf{2}_{35}$) (**c**) measured in $CDCl_3$ at 25 °C.

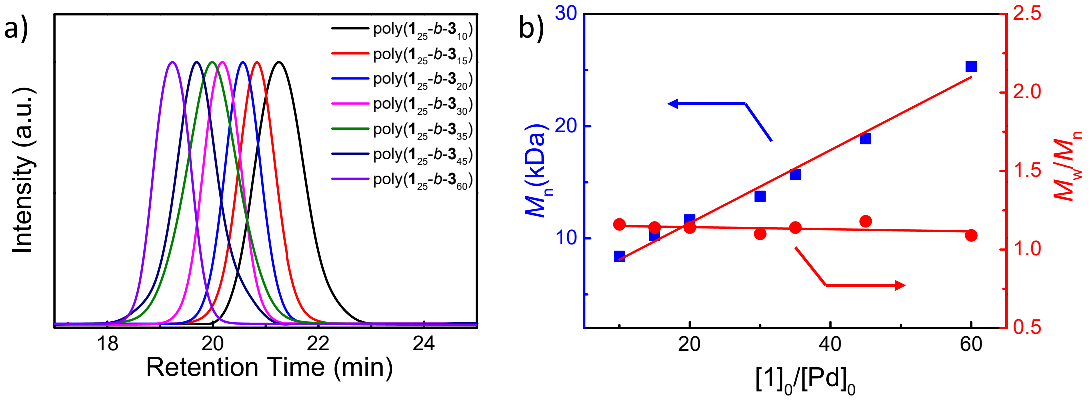

Figure S26. (**a**) SEC chromatograms of poly($\mathbf{1}_{25}$-*b*-$\mathbf{3}_n$) prepared from the copolymerization of monomer **3** with the Pd(II)-terminated poly-$\mathbf{1}_{25}$ as macroinitiator in THF with different initial feed ratios of monomer to catalyst. (**b**) Plots of M_n and M_w/M_n values of poly($\mathbf{1}_m$-*b*-$\mathbf{3}_n$)s measured as a function of the initial feed ratios of monomer **3** to the common macroinitiator, Pd(II)-terminated poly-$\mathbf{1}_{25}$ (M_n = 6.38 kDa, M_w/M_n = 1.17). SEC conditions: temperature = 40 °C; eluent = THF.

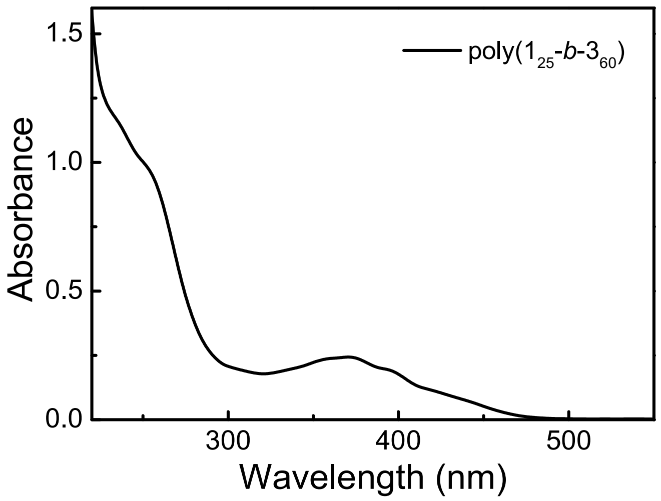

Figure S27. UV-vis absorption spectra of poly($\mathbf{1}_{25}$-*b*-$\mathbf{3}_{60}$) measured in THF at 25 °C (c = 0.03 g/L).

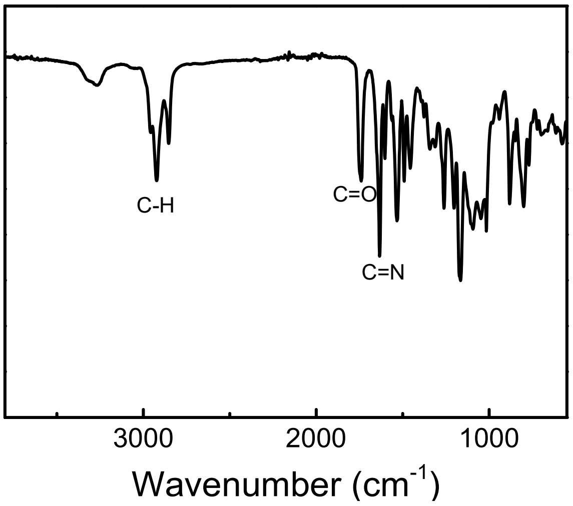

Figure S28. FT-IR spectra of poly($\mathbf{1}_{25}$-*b*-$\mathbf{3}_{60}$) measured at 25 °C using KBr pellets.

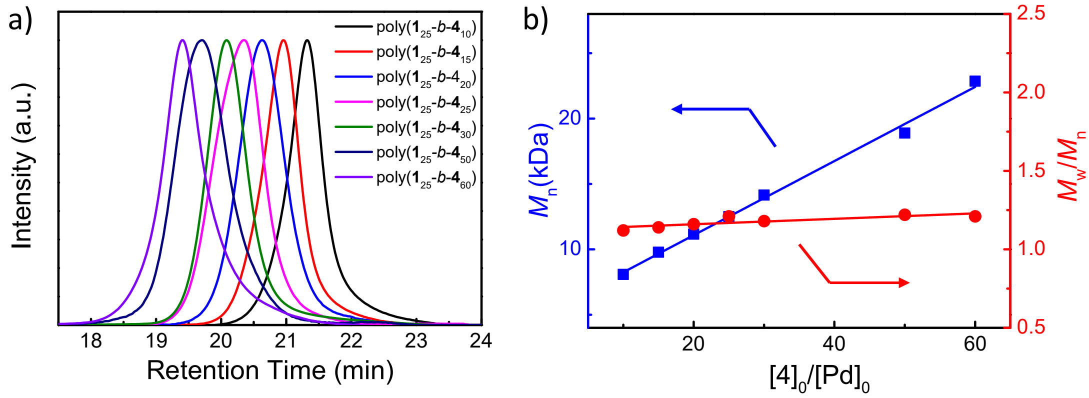

Figure S29. (**a**) SEC chromatograms of poly($\mathbf{1}_{25}$-*b*-$\mathbf{4}_{n}$) prepared from the copolymerization of monomer **3** with the Pd(II)-terminated poly-$\mathbf{1}_{25}$ as macroinitiator in THF with different initial feed ratios of monomer to catalyst. (**b**) Plots of M_n and M_w/M_n values of poly($\mathbf{1}_{m}$-*b*-$\mathbf{4}_{n}$)s measured as a function of the initial feed ratios of monomer **4** to the common macroinitiator, Pd(II)-terminated poly-$\mathbf{1}_{25}$ (M_n = 6.38 kDa, M_w/M_n = 1.17). SEC conditions: temperature = 40 °C; eluent = THF

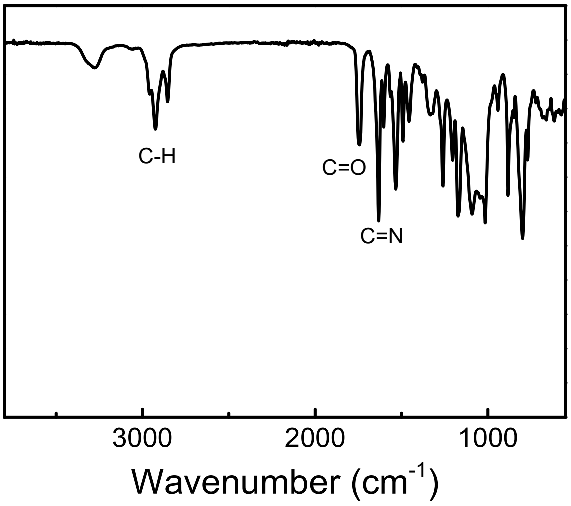

Figure S30. FT-IR spectra of poly($\mathbf{1}_{25}$-*b*-$\mathbf{4}_{30}$) measured at 25 °C using KBr pellets.

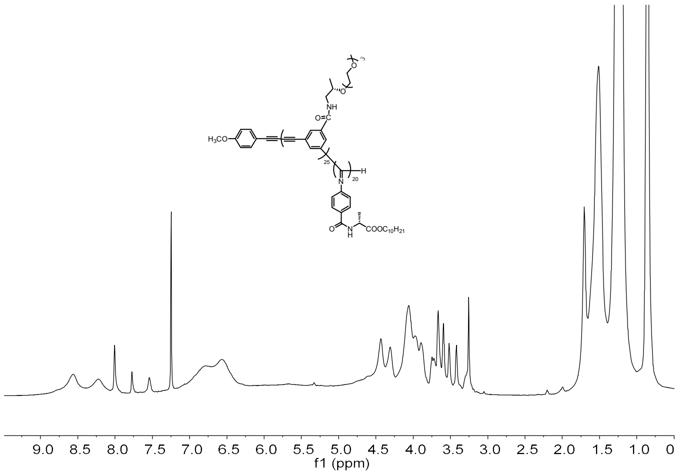

Figure 31. ^{1}H NMR spectra (600 MHz) of PPE-*b*-PPI block copolymer poly($\mathbf{1}_{25}$-*b*-$\mathbf{4}_{20}$) measured in $CDCl_3$ at 25 °C.

© 2018 by the authors. Submitted for possible open access publication under the terms and conditions of the Creative Commons Attribution (CC BY) license (http://creativecommons.org/licenses/by/4.0/).
